# Supplementary material for: Single-cell analysis of a mutant library generated using CRISPR-guided deaminase in human melanoma cells
Source: Commun Biol. 2020 Apr 2;3:154. doi: 10.1038/s42003-020-0888-2 (PMC7118117; doi:10.1038/s42003-020-0888-2)
Supplement: Supplementary file 1 — Supplementary Information [file 42003_2020_888_MOESM1_ESM.pdf]

## Supplementary Figures

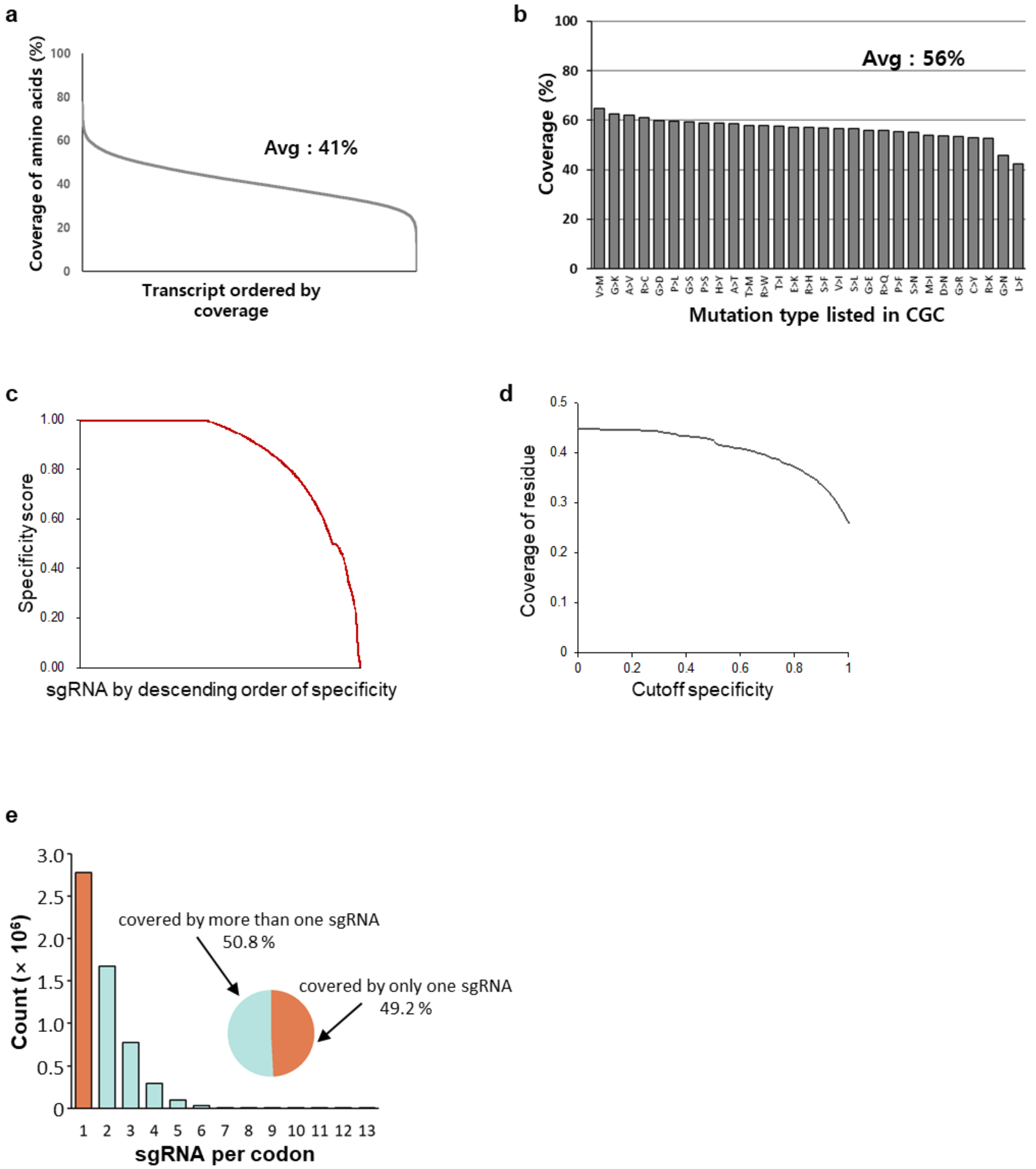


**Supplementary Figure 1 |** Mutations introducible using our system. (**a**) On average, 41% of protein residues per gene isoform were covered by targeting "GG" and "AG" PAMs. (**b**) Introducible disease-related mutations listed in the CGC. (**c**) specificity score for 10,038 sgRNAs designed from 20 randomly selected genes and (**d**) average coverage of residue for all possible isoforms depending on an applied cutoff of specificity score binned by intervals of 0.01. (**e**) Number and distribution of sgRNAs, indicating that more than 50% of codons can be covered by more than one sgRNA.

**a**

| | PAM | | | | | | | | | | | | | Arg | | | Gly | | | Glu | | | |
|---|---|---|---|---|---|---|---|---|---|---|---|---|---|---|---|---|---|---|---|---|---|---|---|
| | C | C | T | A | G | T | C | A | A | C | T | C | C | C | G | T | G | G | G | G | A | G | A |
| **A** | 0.00 | 0.02 | 0.00 | **100.00** | 0.03 | 0.00 | 0.00 | **100.00** | **99.99** | 0.00 | 0.00 | 0.00 | 0.00 | 0.01 | 4.27 | 0.00 | 51.59 | 76.04 | 72.21 | 82.84 | **99.99** | 43.19 | **100.00** |
| **C** | **99.99** | **99.98** | 0.01 | 0.00 | 0.00 | 0.01 | **99.99** | 0.00 | 0.00 | **99.99** | 0.01 | **99.99** | **99.99** | **99.98** | 0.00 | 0.01 | 3.73 | 0.71 | 0.04 | 3.67 | 0.00 | 0.05 | 0.00 |
| **G** | 0.00 | 0.00 | 0.00 | 0.00 | **99.97** | 0.00 | 0.00 | 0.00 | 0.00 | 0.00 | 0.00 | 0.00 | 0.00 | 0.00 | **95.73** | 0.00 | **44.65** | **22.99** | **23.66** | **13.45** | 0.00 | **56.39** | 0.00 |
| **T** | 0.00 | 0.00 | **99.99** | 0.00 | 0.00 | **99.99** | 0.00 | 0.00 | 0.00 | 0.01 | **99.99** | 0.00 | 0.01 | 0.01 | 0.00 | **99.99** | 0.04 | 0.26 | 4.09 | 0.04 | 0.00 | 0.37 | 0.00 |

**b**

| Initial | |
|---|---|
| processivity | percent |
| CGTGGGGAGA | 93.55 |
| CGTAAAAAAA | 2.38 |
| CGTAAAAAGA | 1.46 |
| CGTGAAAAGA | 0.67 |

| DMSO | |
|---|---|
| processivity | percent |
| CGTGGGGAGA | 86.11 |
| CGTAAAAAAA | 5.23 |
| CGTAAAAAGA | 3.38 |
| CGTGAAAAGA | 0.91 |

| Vemurafenib_1N | |
|---|---|
| processivity | percent |
| CGTAAAAAAA | 27.44 |
| CGTAAAAAGA | 18.15 |
| CGTGAAAAGA | 13.82 |
| CGTGGGGAGA | 12.72 |

| Vemurafenib_2N | |
|---|---|
| processivity | percent |
| CGTGAAAAGA | 28.66 |
| CGTGGGGAGA | 17.82 |
| CGTAAAAAAA | 14.29 |
| CGTAAAAAGA | 9.79 |

| Vemurafenib_3N | |
|---|---|
| processivity | percent |
| CGTAAAAAAA | 24.35 |
| CGTGGGGAGA | 17.55 |
| CGTGAAAAGA | 11.83 |
| CGTAAAAAGA | 10.66 |

**Supplementary Figure 2 |** Conversions of C were observed within the 'activity window,' and processive conversions were observed. (**a**) Percentage of sequencing reads with the corresponding base is shown along with the sequence of the protospacer of the sgRNA. The plot is shown for the protein-coding strand, which is the non-target strand of the sgRNA; therefore, guanine was considered the target instead of cytosine. (**b**) Processive deamination was observed. The PAM-proximal 10 nt were examined for processivity. Convertible guanine is underlined, and converted adenine is shown in red font. To improve the confidence of the experimental enrichment for vemurafenib resistance, cells transduced by the virus in a single experiment were split into three sub-pools. Cell sub-pools were each treated with vemurafenib in triplicate.

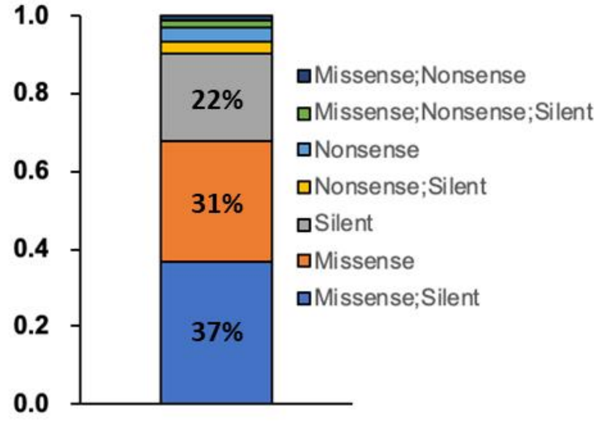


**Supplementary Figure 3 |** Fraction of each introducible type of mutation using the 420-sgRNA library.

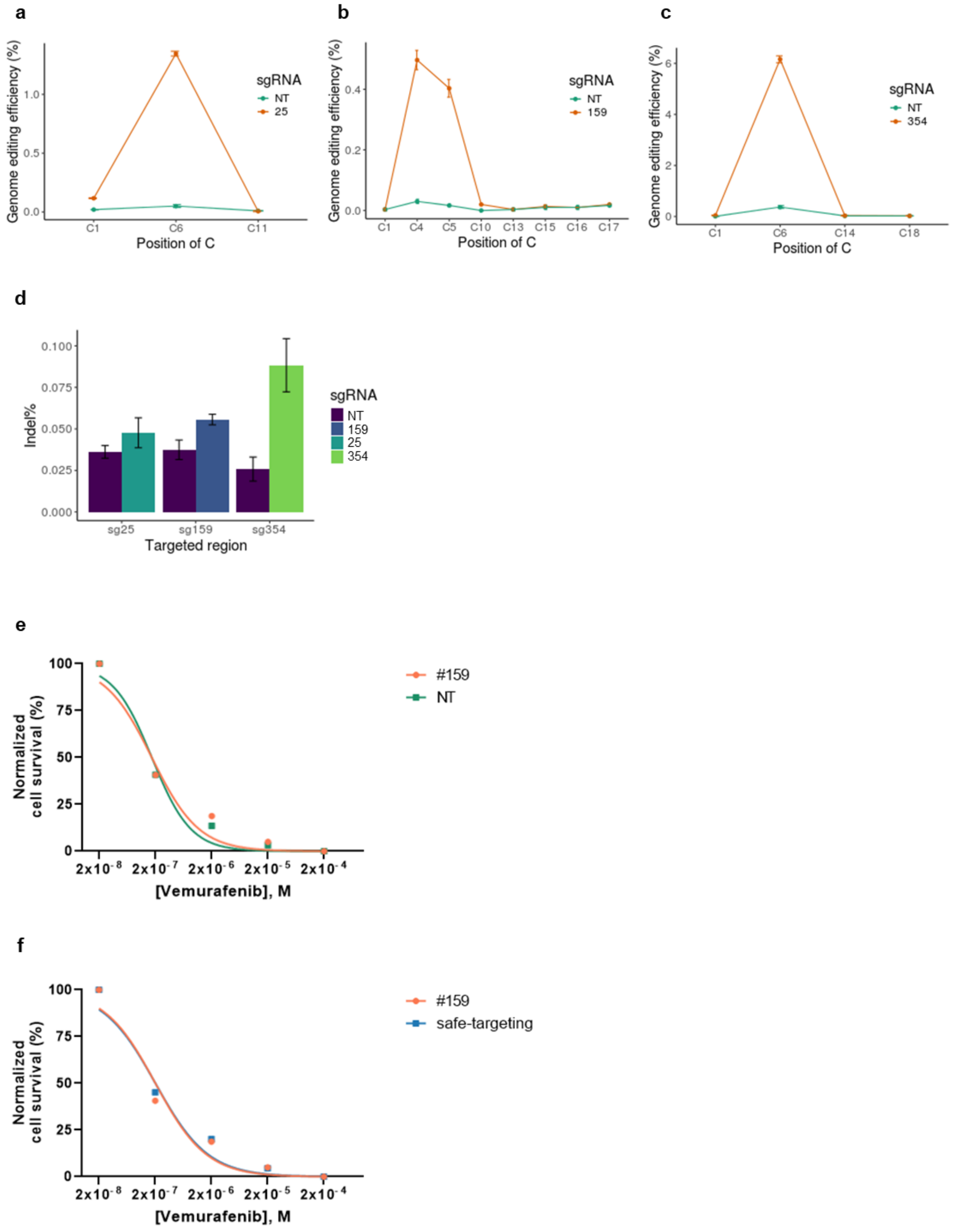


**Supplementary Figure 4 |** Singleplex experiments to test a library of sgRNAs. (**a-c**) Efficiency of cytosine substitution according to position in the protospacer of the #25, #159, and #354 sgRNAs, respectively. Error bars represent the standard error of mean from three independent experiments. (**d**) Bar graph showing indel frequency in cells individually transduced by #25, #159, and #354 sgRNA. Error bars represent the standard error of mean from three independent experiments.

Vemurafenib dose response curves for (**e**) cells transduced with #159 sgRNA or non-targeting sgRNA ($F_{1,16} = 0.1275$, $p = 0.7258$, extra sum-of-squares F test between estimated logIC50, n = 2 replicates) and (**f**) cells transduced with #159 sgRNA or safe-targeting sgRNA ($F_{1,16} = 0.4238$, $p = 0.5243$, extra sum-of-squares F test between estimated logIC50, n = 2 replicates).

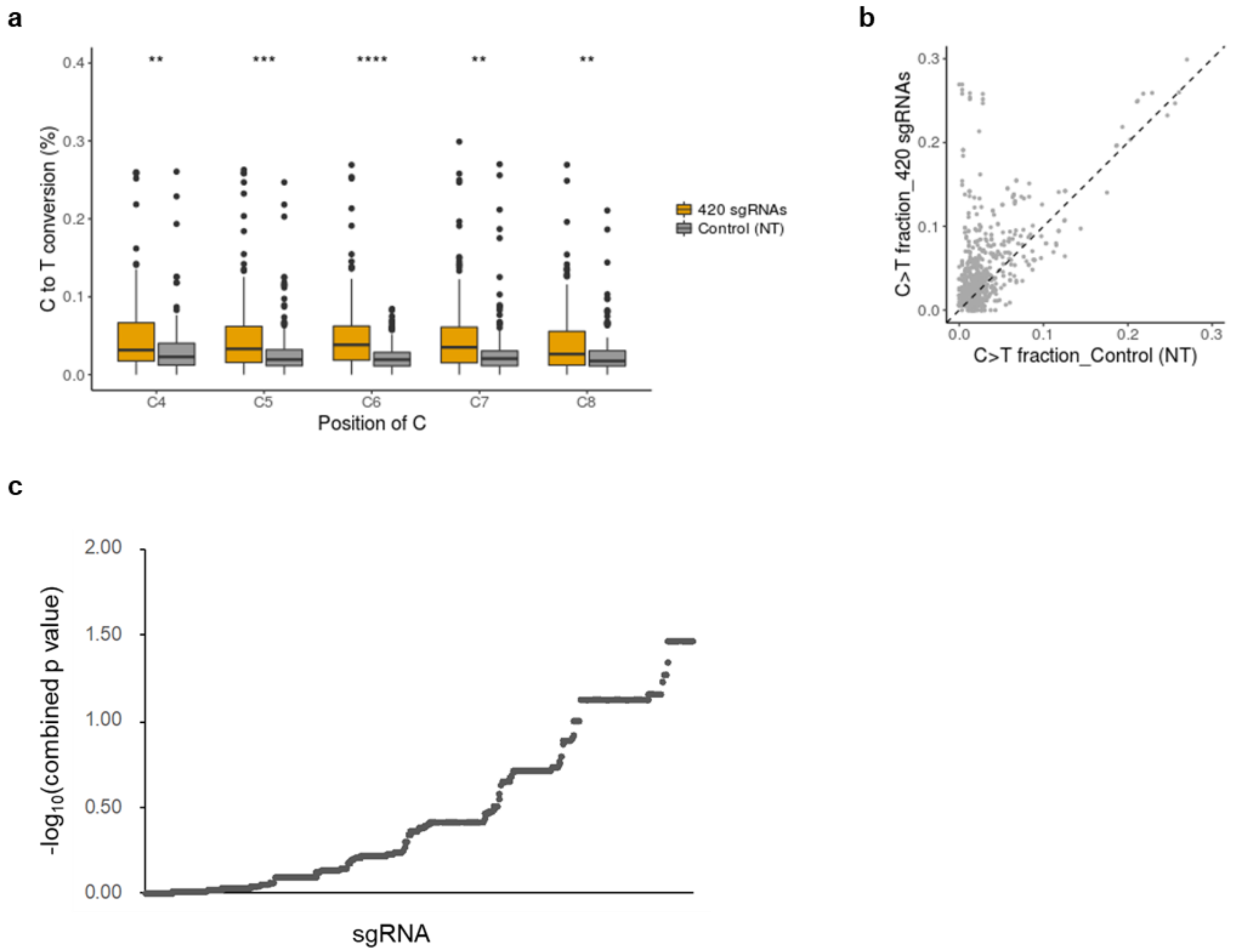


**Supplementary Figure 5 |** Investigation of C to T conversion from 420 sgRNA-treated cells before drug selection. (**a**) Boxplot showing C to T conversion frequency within the 'activity window' for 420 sgRNAs. All p-values determined by two-sided Wilcoxon rank-sum test; ****$p < 0.0001$, ***$p < 0.001$, **$p < 0.01$, ns = not significant ($p > 0.05$). The center line corresponds to the median; the edges of the box correspond to the first and third quartiles; the whiskers represent 1.5× the interquartile range; and the black dots indicate outliers. (**b**) Plot of average C to T fraction of each “C” within the ‘activity window’. (**c**) Statistical significance of the C to T conversion ratio. Difference of C to T conversion between cells treated with 420 sgRNA and non-targeting sgRNA was tested with the one-sided Mann-Whitney U test and corrected using Benjamini-Hochberg method. P-values for each sgRNA were aggregated using Fisher’s method. Data for 420 sgRNA were produced using two independent replicate experiments, and data for non-targeting (NT) were produced using three independent replicates experiments.

**a**

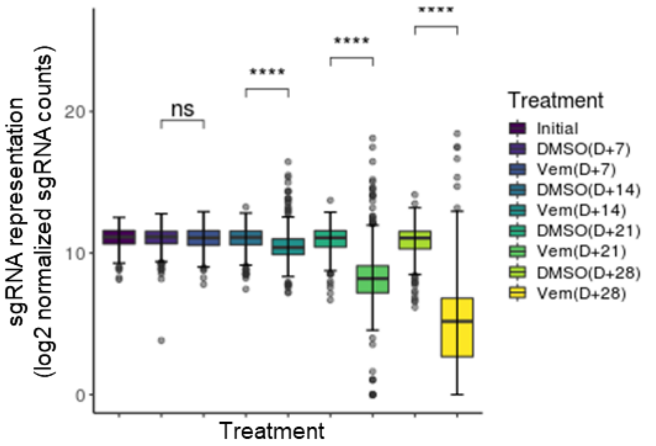


**b**

DMSO (D+28)

| | PAM | | | | | | | | Glu | | | | | | Lys | | | | | | | | |
|---|---|---|---|---|---|---|---|---|---|---|---|---|---|---|---|---|---|---|---|---|---|---|---|
| | C | C | C | G | T | G | G | G | G | A | G | A | T | C | A | A | G | C | T | C | T | G | T |
| A | 0.00 | 0.01 | 0.00 | 0.02 | 0.01 | 0.01 | 0.01 | 0.02 | 0.03 | 99.98 | 0.04 | 99.98 | 0.01 | 0.00 | 99.98 | 99.99 | 0.03 | 0.00 | 0.00 | 0.00 | 0.01 | 0.01 | 0.01 |
| C | 99.99 | 99.99 | 99.98 | 0.00 | 0.01 | 0.00 | 0.00 | 0.00 | 0.00 | 0.00 | 0.01 | 0.00 | 0.01 | 99.93 | 0.00 | 0.00 | 0.01 | 99.99 | 0.02 | 99.98 | 0.02 | 0.00 | 0.01 |
| G | 0.00 | 0.00 | 0.00 | 99.97 | 0.00 | 99.99 | 99.98 | 99.97 | 99.96 | 0.01 | 99.95 | 0.01 | 0.00 | 0.01 | 0.01 | 0.01 | 99.96 | 0.00 | 0.00 | 0.00 | 0.00 | 99.99 | 0.00 |
| T | 0.01 | 0.01 | 0.02 | 0.00 | 99.98 | 0.00 | 0.00 | 0.00 | 0.01 | 0.01 | 0.00 | 0.01 | 99.97 | 0.06 | 0.01 | 0.00 | 0.00 | 0.00 | 99.98 | 0.02 | 99.97 | 0.00 | 99.98 |

Vem (D+28)

| | PAM | | | | | | | | Glu | | | | | | Lys | | | | | | | | |
|---|---|---|---|---|---|---|---|---|---|---|---|---|---|---|---|---|---|---|---|---|---|---|---|
| | C | C | C | G | T | G | G | G | G | A | G | A | T | C | A | A | G | C | T | C | T | G | T |
| A | 0.00 | 0.00 | 0.00 | 0.02 | 0.02 | 0.03 | 0.38 | 6.55 | 70.47 | 99.98 | 14.62 | 99.97 | 0.01 | 0.00 | 99.98 | 99.99 | 23.11 | 0.00 | 0.00 | 0.00 | 0.01 | 0.01 | 0.01 |
| C | 99.99 | 99.99 | 99.97 | 0.00 | 0.01 | 0.00 | 0.00 | 0.00 | 0.00 | 0.00 | 0.05 | 0.00 | 0.00 | 99.99 | 0.00 | 0.00 | 0.09 | 99.99 | 0.03 | 99.99 | 0.02 | 0.00 | 0.01 |
| G | 0.00 | 0.00 | 0.00 | 99.97 | 0.00 | 99.97 | 99.61 | 93.44 | 29.47 | 0.01 | 85.33 | 0.01 | 0.00 | 0.00 | 0.01 | 0.01 | 76.69 | 0.00 | 0.00 | 0.00 | 0.00 | 99.99 | 0.00 |
| T | 0.01 | 0.01 | 0.03 | 0.00 | 99.97 | 0.00 | 0.00 | 0.01 | 0.06 | 0.00 | 0.01 | 0.01 | 99.98 | 0.01 | 0.00 | 0.00 | 0.11 | 0.01 | 99.97 | 0.00 | 99.97 | 0.00 | 99.98 |

**c**

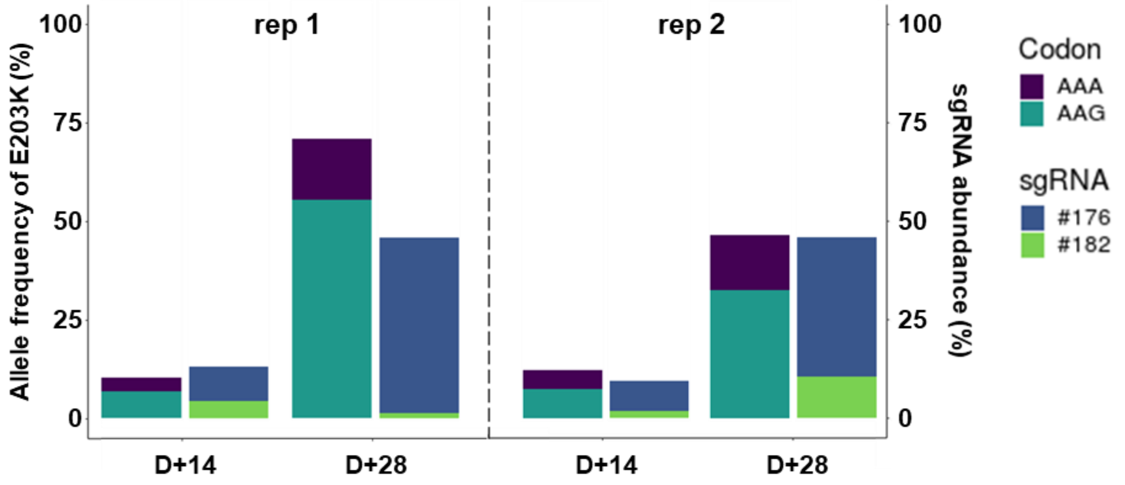


**d**

DMSO (D+28)

| | PAM | | | | | | | | Glu | | | | | | Lys | | | | | | | | |
|---|---|---|---|---|---|---|---|---|---|---|---|---|---|---|---|---|---|---|---|---|---|---|---|
| | C | C | C | G | T | G | G | G | G | A | G | A | T | C | A | A | G | C | T | C | T | G | T |
| A | 0.00 | 0.00 | 0.00 | 0.03 | 0.01 | 0.02 | 0.02 | 0.02 | 0.05 | 99.99 | 0.05 | 99.98 | 0.02 | 0.00 | 99.99 | 99.98 | 0.04 | 0.00 | 0.00 | 0.00 | 0.01 | 0.02 | 0.01 |
| C | 99.99 | 99.98 | 99.97 | 0.00 | 0.02 | 0.00 | 0.00 | 0.00 | 0.00 | 0.00 | 0.00 | 0.00 | 0.00 | 99.97 | 0.00 | 0.00 | 0.01 | 99.99 | 0.01 | 99.97 | 0.03 | 0.00 | 0.00 |
| G | 0.00 | 0.00 | 0.00 | 99.97 | 0.02 | 99.98 | 99.98 | 99.97 | 99.93 | 0.01 | 99.94 | 0.01 | 0.00 | 0.00 | 0.00 | 0.02 | 99.96 | 0.00 | 0.01 | 0.01 | 0.01 | 99.98 | 0.00 |
| T | 0.01 | 0.02 | 0.03 | 0.00 | 99.94 | 0.00 | 0.00 | 0.00 | 0.02 | 0.00 | 0.00 | 0.00 | 99.98 | 0.03 | 0.00 | 0.00 | 0.00 | 0.01 | 99.97 | 0.02 | 99.96 | 0.00 | 99.98 |

Vem (D+28)

| | PAM | | | | | | | | Glu | | | | | | Lys | | | | | | | | |
|---|---|---|---|---|---|---|---|---|---|---|---|---|---|---|---|---|---|---|---|---|---|---|---|
| | C | C | C | G | T | G | G | G | G | A | G | A | T | C | A | A | G | C | T | C | T | G | T |
| A | 0.00 | 0.00 | 0.00 | 0.03 | 0.02 | 0.02 | 1.60 | 6.60 | 48.07 | 99.97 | 14.88 | 99.97 | 0.02 | 0.00 | 99.95 | 99.99 | 9.15 | 0.00 | 0.00 | 0.00 | 0.00 | 0.00 | 0.01 |
| C | 99.99 | 99.98 | 99.98 | 0.00 | 0.00 | 0.00 | 0.00 | 0.00 | 0.06 | 0.00 | 0.38 | 0.00 | 0.01 | 99.99 | 0.00 | 0.00 | 0.32 | 100.00 | 0.01 | 100.00 | 0.03 | 0.00 | 0.01 |
| G | 0.00 | 0.00 | 0.00 | 99.97 | 0.00 | 99.97 | 98.40 | 93.33 | 51.81 | 0.01 | 84.72 | 0.02 | 0.00 | 0.00 | 0.04 | 0.01 | 90.39 | 0.00 | 0.03 | 0.00 | 0.00 | 100.00 | 0.00 |
| T | 0.01 | 0.02 | 0.01 | 0.00 | 99.98 | 0.00 | 0.00 | 0.07 | 0.05 | 0.02 | 0.02 | 0.01 | 99.96 | 0.01 | 0.01 | 0.00 | 0.15 | 0.00 | 99.96 | 0.00 | 99.97 | 0.00 | 99.98 |

**Supplementary Figure 6 |** Introduction and functional screening of multiple mutations using population analysis. (**a**) Box plot showing the distribution of reads from individual sgRNAs according to time (initial, D+7, D+14, D+21, and D+28) and condition (DMSO, Vem) in replicate 2. All p-values determined by two-sided Wilcoxon rank-sum test, ****$p < 0.0001$. ns: not significant ($p > 0.05$) ($n = 420$ individual sgRNAs). The center line corresponds to the median; the edges of the box correspond to the first and third quartiles; the whiskers represent 1.5× the interquartile range; and the black dots indicate outliers. (**b**) Percentage of sequencing reads with the corresponding base is shown along with the sequence of the protospacer of the #176 sgRNA in replicate 1. The plot is shown for the protein-coding strand, which is the non-target strand of the sgRNA; therefore, guanine was considered the target instead of cytosine. (**c**) Bar graph showing the allele frequency of the E203K clone and abundance of sgRNA-introduced E203K mutation in *MAP2K1* from experiment replicates 1 and 2. (**d**) Percentage of sequencing reads with the corresponding base is shown along with the sequence of the protospacer of the #176 sgRNA in replicate 2. The plot is shown for the protein-coding strand, which is the non-target strand of the sgRNA; therefore, guanine was considered the target instead of cytosine.

a

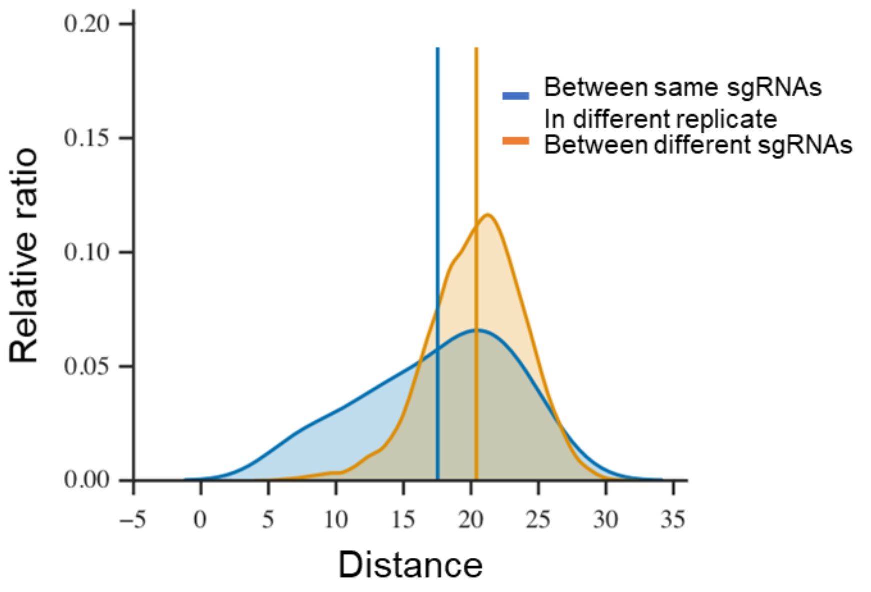


b

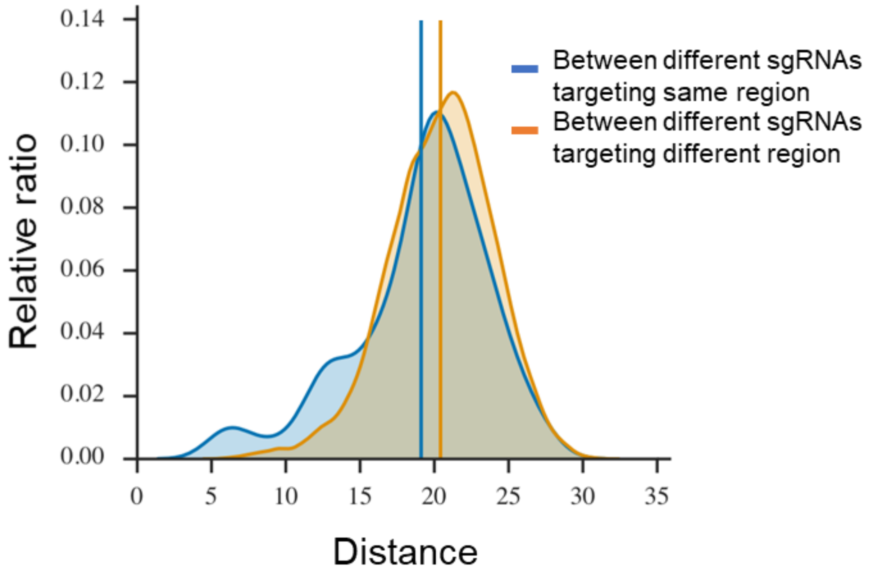


**Supplementary Figure 7 |** Similarity between sgRNAs. (**a**) Distribution of distance between the same sgRNA in different replicates (blue) and different sgRNAs (red). (**b**) Distribution of distance between different sgRNAs targeting the same region (blue) and different sgRNAs targeting different regions (red).

**a**

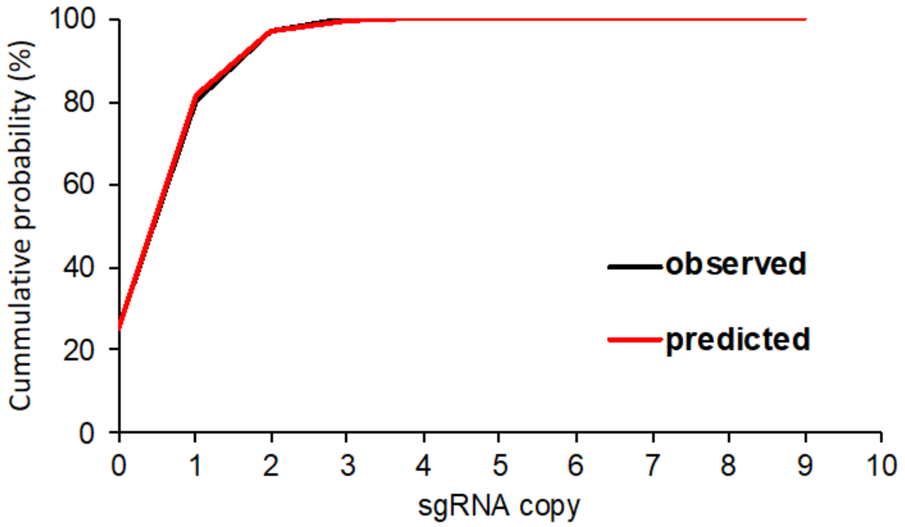


**b**

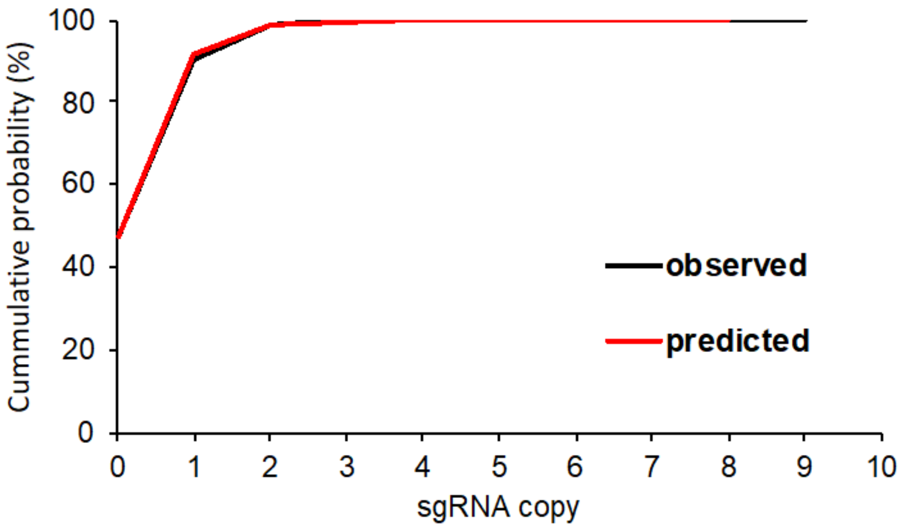


**Supplementary Figure 8 |** Distribution of observed sgRNA copy number per cell (black) and the predicted value from the maximum likelihood estimate (red) for replicate 1 (**a**) and replicate 2 (**b**) samples.

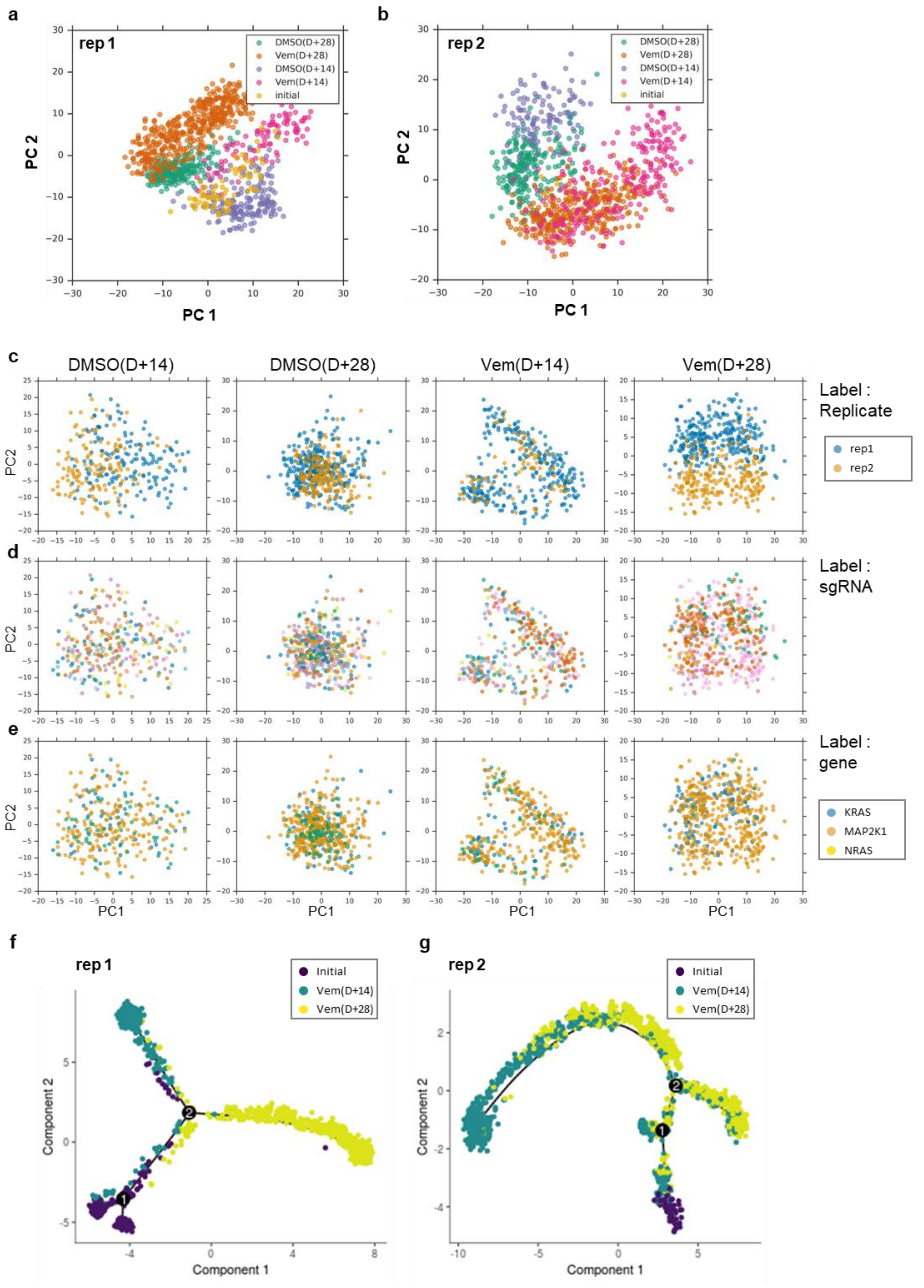


**Supplementary Figure 9 |** Global clustering across the different treatment periods. (**a, b**) PCA of transcriptomes for cells from the Initial, D+14, and D+28 data in replicate 1 (**a**) and replicate 2 (**b**). (**c-e**) PCA of transcriptomes for cells from DMSO(D+14), DMSO(D+28), Vem(D+14), and Vem(D+28). Cells were labeled according to replicate (**c**), sgRNA (**d**), and gene (**e**). (**f, g**) Trajectory

analysis using Monocle 2. The trajectories of Vem(D+14) and Vem(D+28) branched to different clusters in both replicates, and no progression according to treatment period was observed.

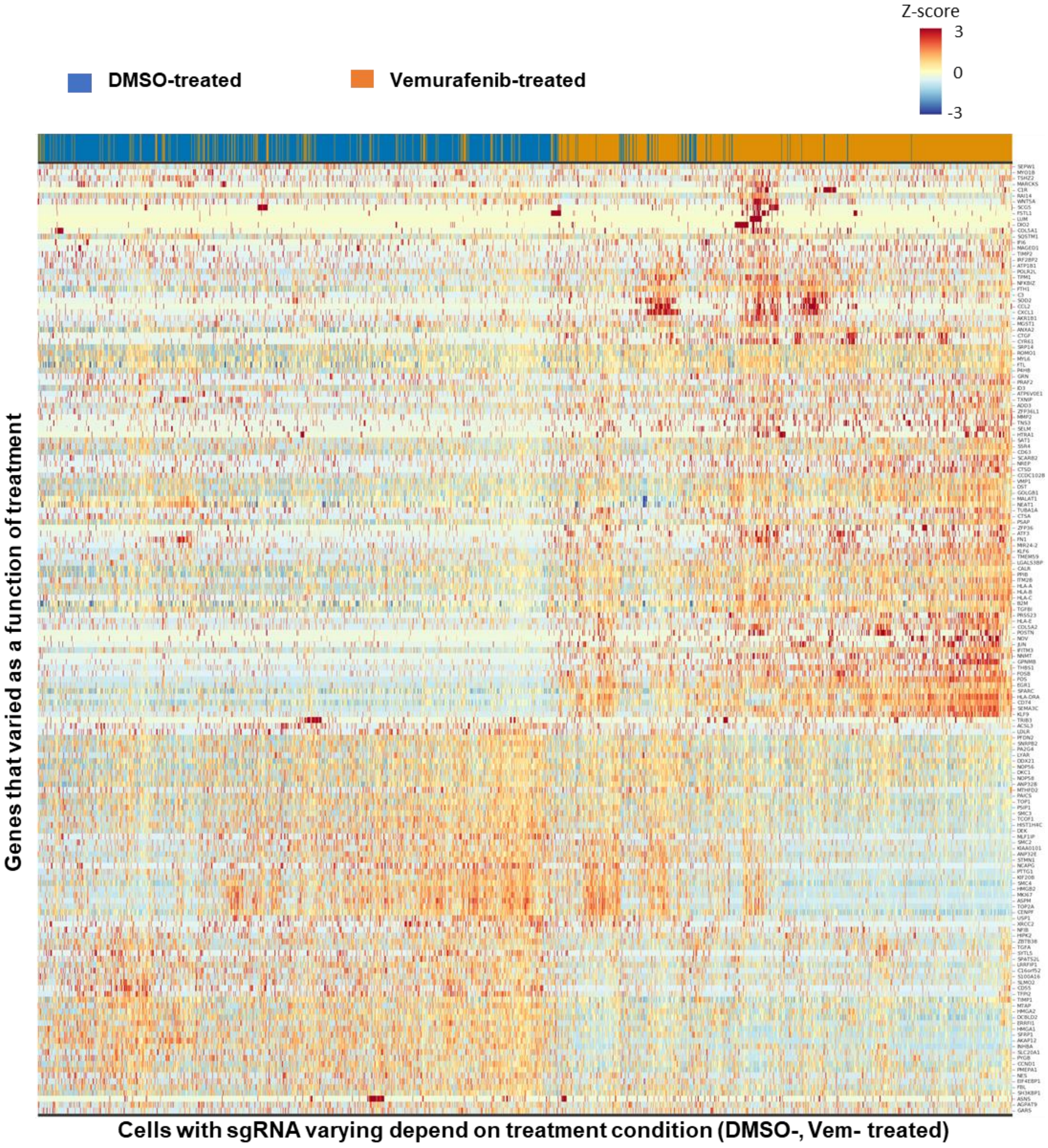


**Supplementary Figure 10 |** Average expression of genes, which were observed to vary with vemurafenib treatment, according to individual sgRNA. Each row represents genes and each column represents sgRNA. Color scale indicates the standard deviation of gene expression from the mean expression value, with red indicating high expression and blue indication low expression.

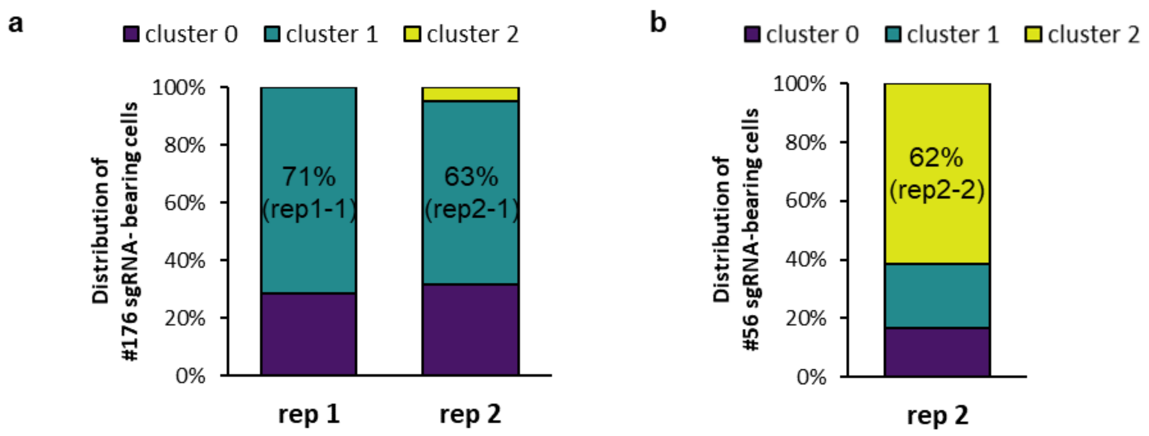


**Supplementary Figure 11 |** (**a**) Distribution of cells with the #176 sgRNA across clusters. Boxplot shows the fraction of #176 sgRNA located in each cluster. (**b**) Boxplot shows the fraction of #56 sgRNA located in each cluster.

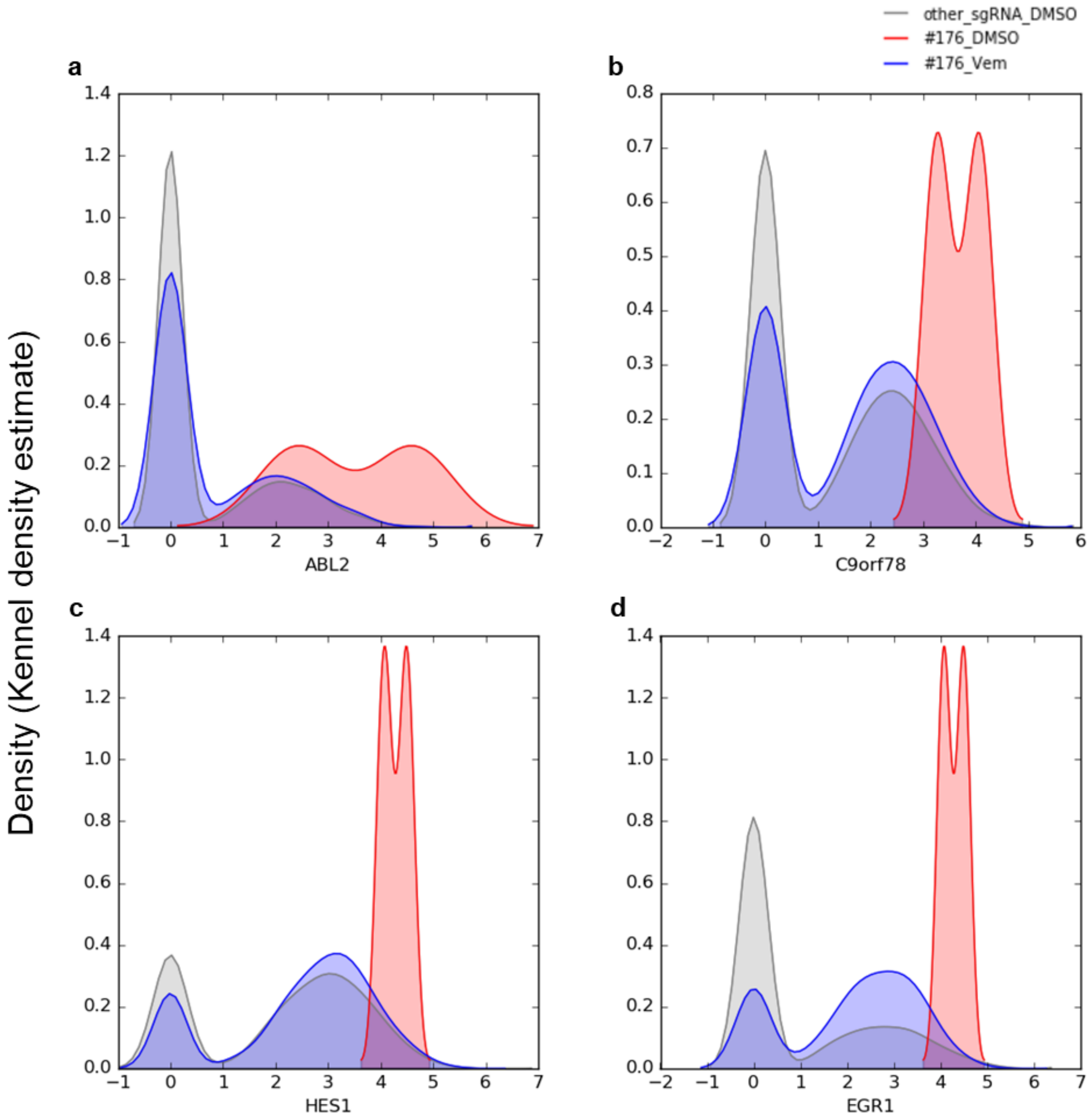


**Supplementary Figure 12 |** Expression of upregulated genes (**a**: *ABL2*, **b**: *C9orf78*, **c**: *HES1*, **d**: *EGR1*) in DMSO-treated cells with #176 sgRNA.

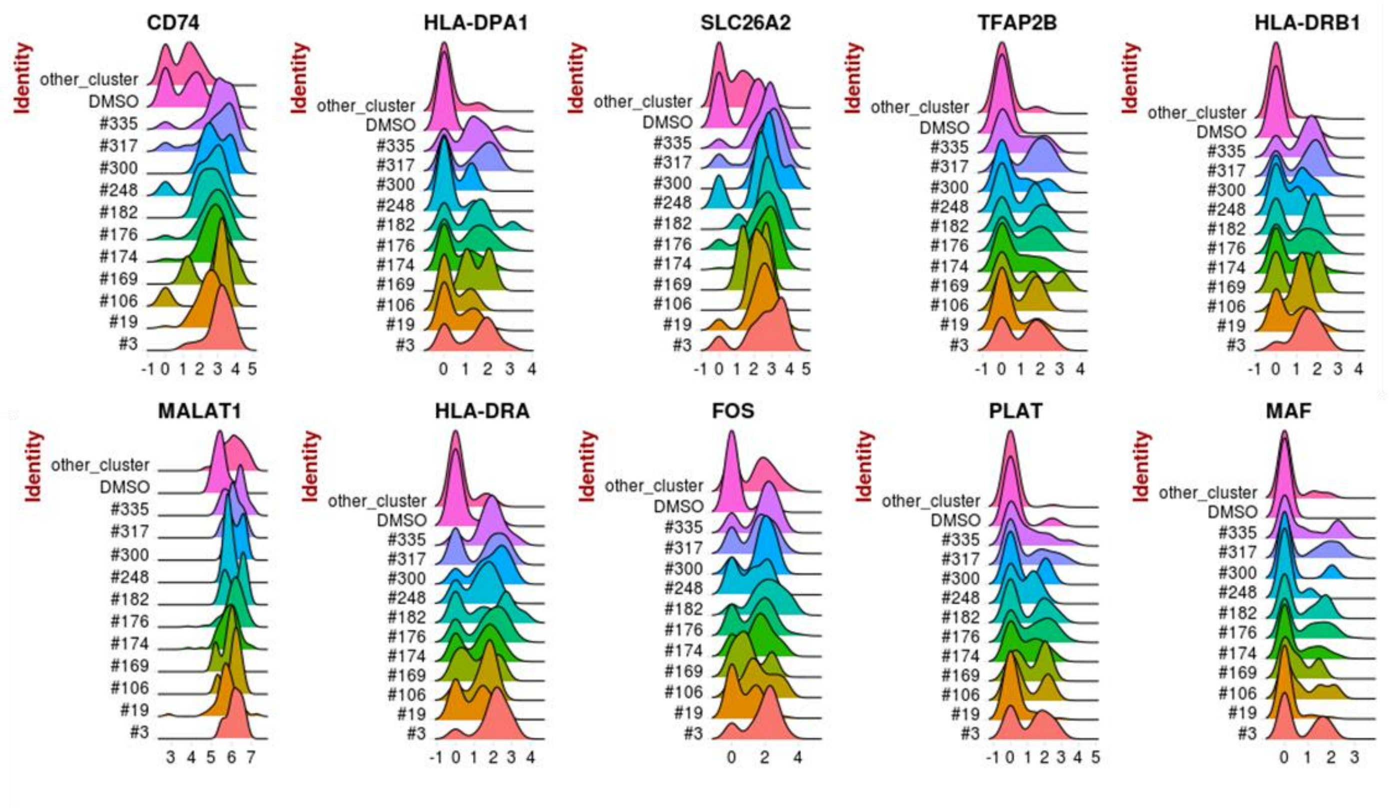


**Supplementary Figure 13 |** Visualization of single-cell expression of signature genes. Vem(D+28) cells in rep1-0 and rep1-2 were grouped into the "other_cluster" category, and DMSO(D+28) cells were grouped into the "DMSO" category. Cells in the rep1-1 cluster were plotted individually according to sgRNAs for which the number of cells was >5.

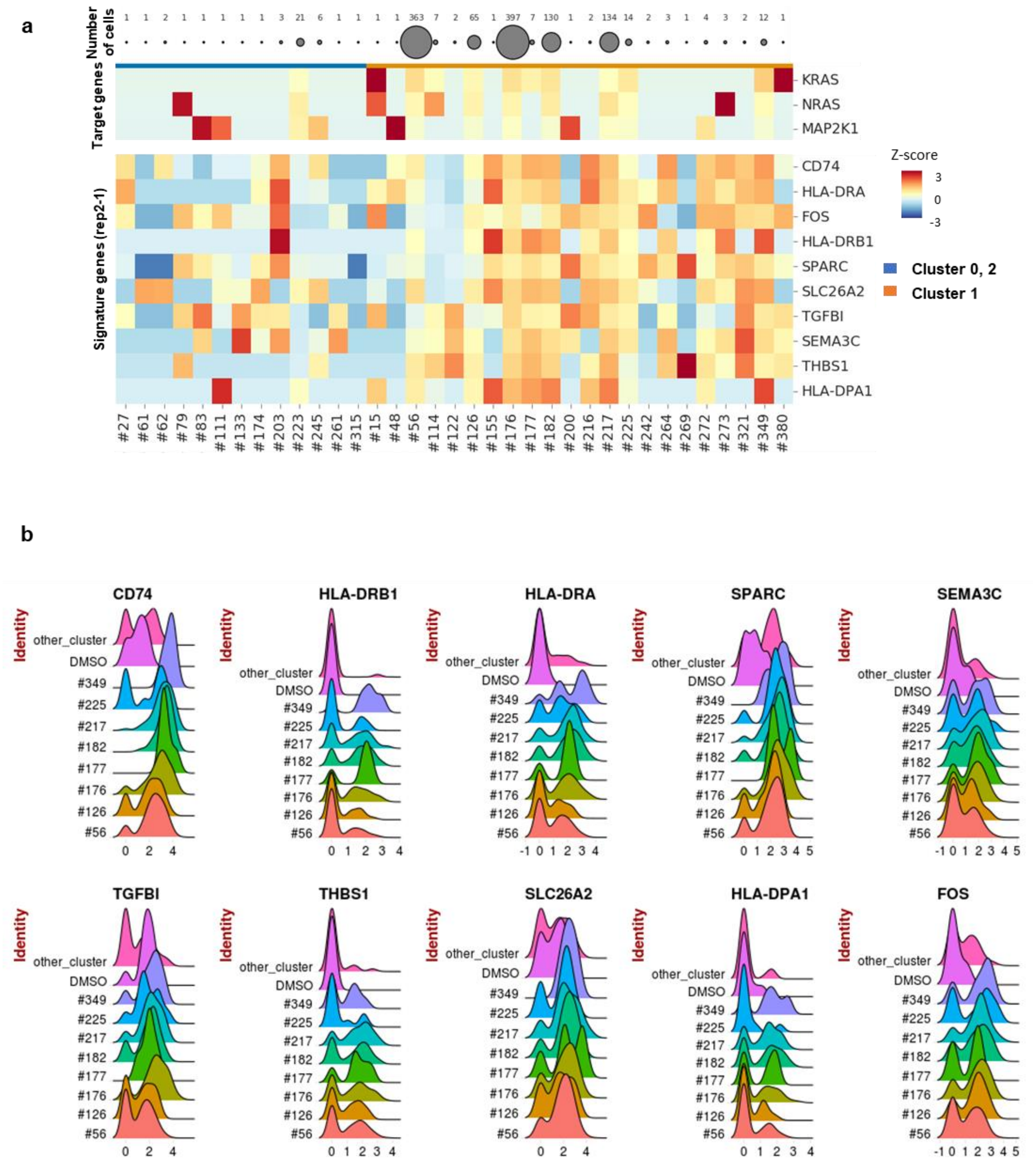


**Supplementary Figure 14 |** Profile of the transcription of signature genes in clusters composed primarily of the #176 sgRNA in replicate 2. (**a**) Average expression of signature genes according to individual sgRNA. Each row represents one of the signature genes, and each column represents cells bearing the sgRNA. Cells from Vem(D+28) in other clusters or Vem(D+28) in rep2-1 are arranged from left to right. Color scale indicates the standard deviation of gene expression from the mean expression value, with red indicating high expression and blue indicating low expression. (**b**) Visualization of expression of signature genes in single cells. Vem(D+28) cells in rep2-0 and rep2-2 were grouped into the "other_cluster" category, and DMSO(D+28) cells were grouped into the "DMSO" category. Cells in the rep2-1 cluster were plotted individually according to sgRNAs for which the number of cells was >5.

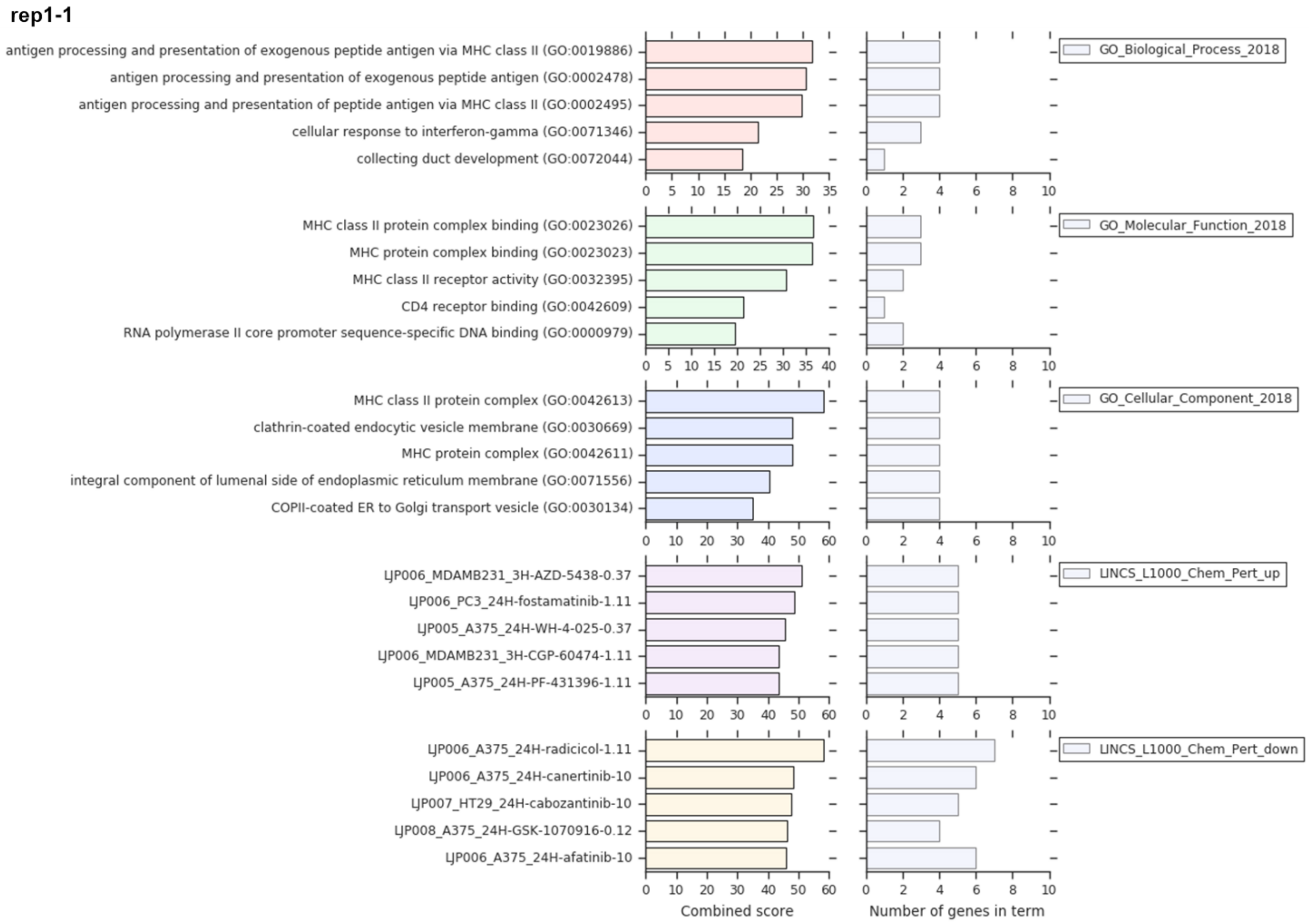


**Supplementary Figure 15 |** Gene set analysis of marker genes in cluster rep1-1 (left panels) and the number of genes overlapping with each term (right panels).

**rep2-1**

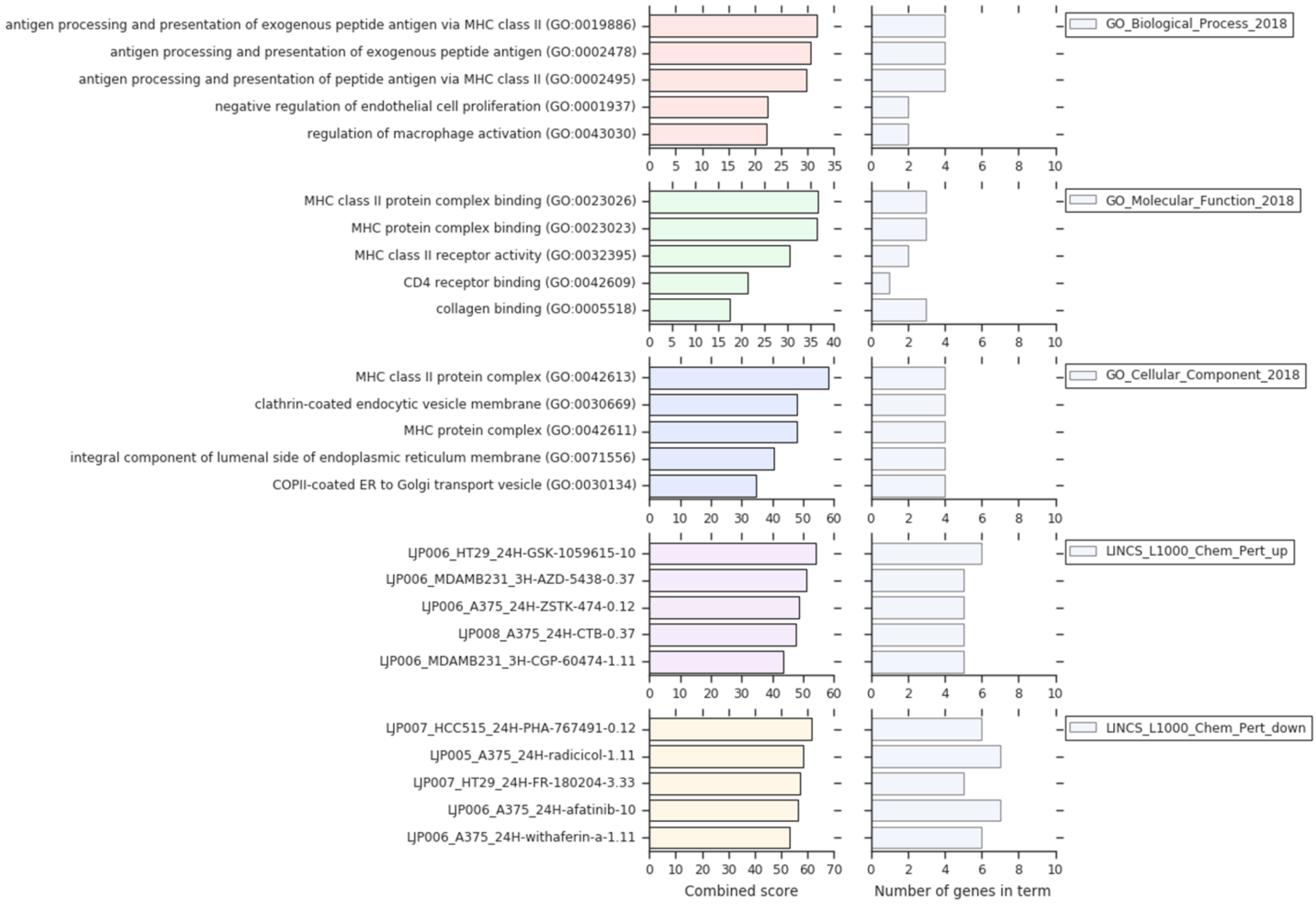


**Supplementary Figure 16 |** Gene set analysis of marker genes in cluster rep2-1 (left panels) and the number of genes overlapping with each term (right panels).

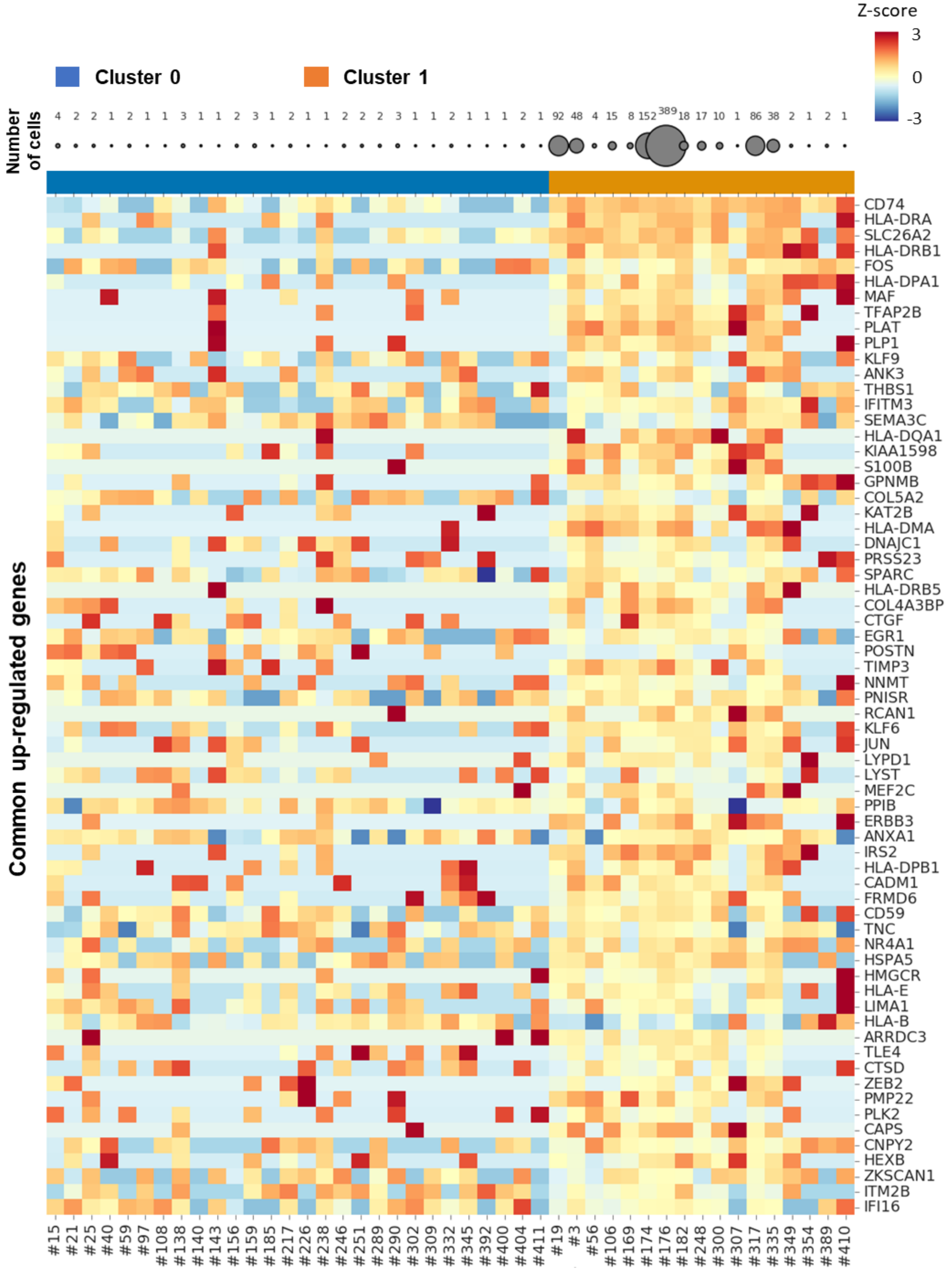


**Supplementary Figure 17 |** Average expression of signature genes according to individual sgRNA. Each row represents one of the common 66 up-regulated genes, and each column represents cells bearing the sgRNA. Cells from Vem(D+28) in other clusters or Vem(D+28) in rep1-1 are arranged from left to right. Color scale indicates the standard deviation of gene expression from the mean expression value, with red indicating high expression and blue indicating low expression.

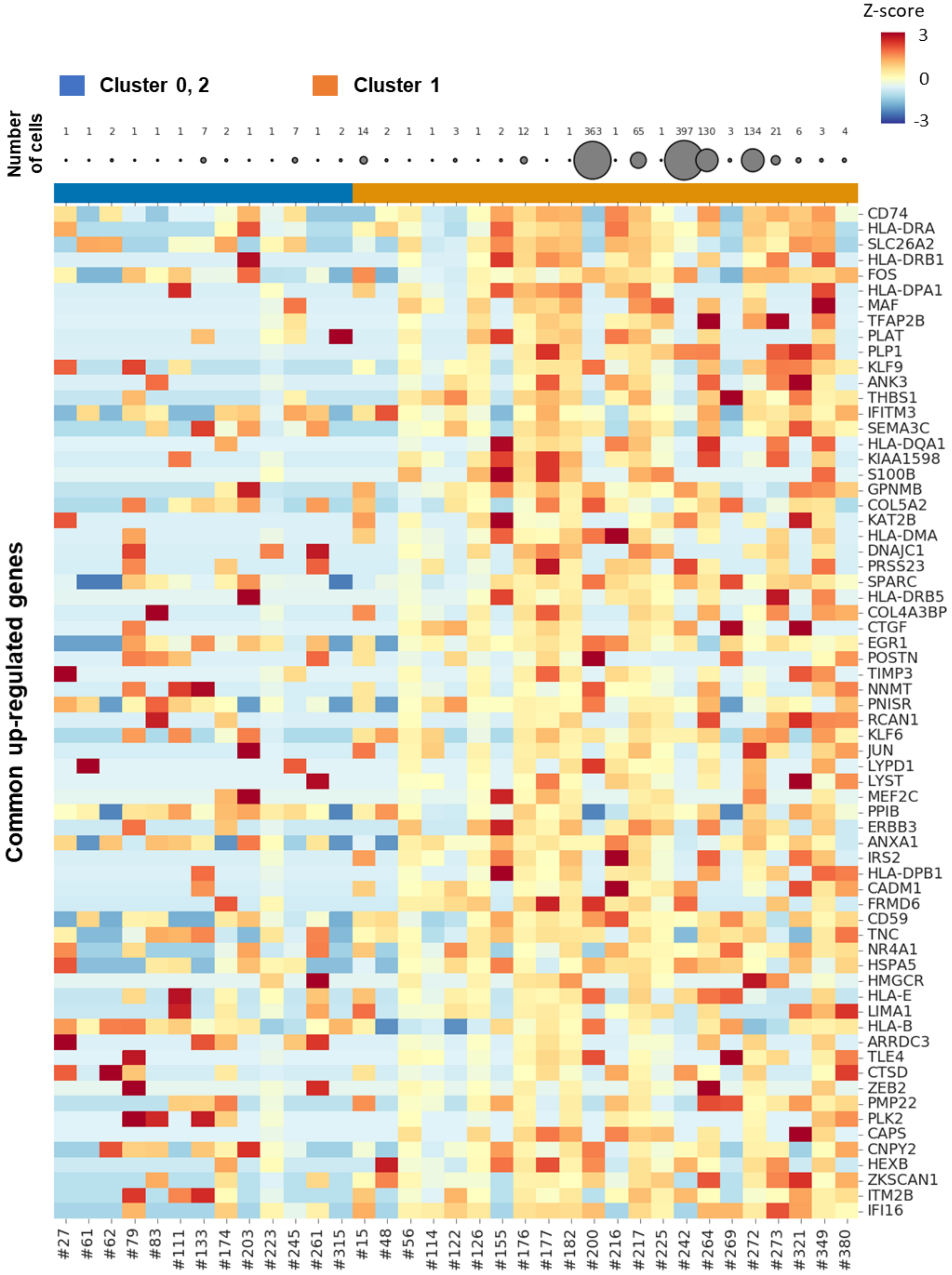


**Supplementary Figure 18 |** Average expression of signature genes according to individual sgRNA. Each row represents one of the common 66 up-regulated genes, and each column represents cells bearing the sgRNA. Cells from Vem(D+28) in other clusters or Vem(D+28) in rep2-1 are arranged from left to right. Color scale indicates the standard deviation of gene expression from the mean expression value, with red indicating high expression and blue indicating low expression.

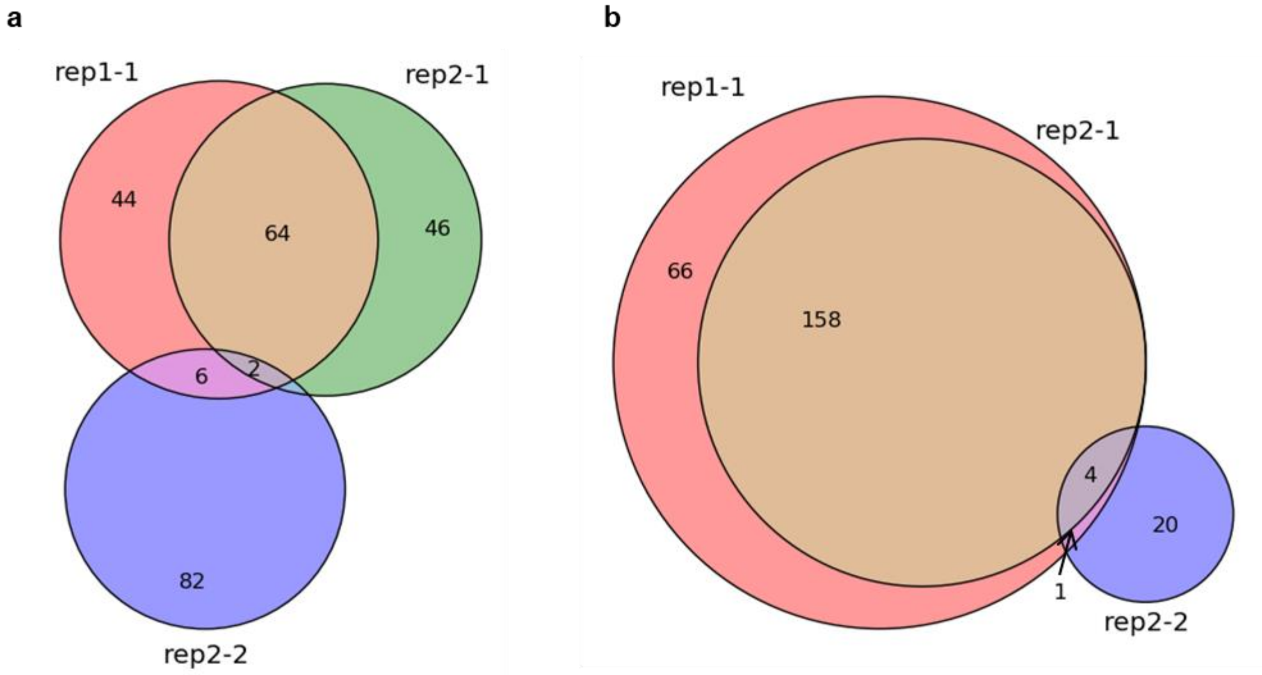


**Supplementary Figure 19 |** Venn diagram of upregulated (**a**) and downregulated (**b**) marker genes with p-values <0.05. The number of genes in each set and intersection are shown.

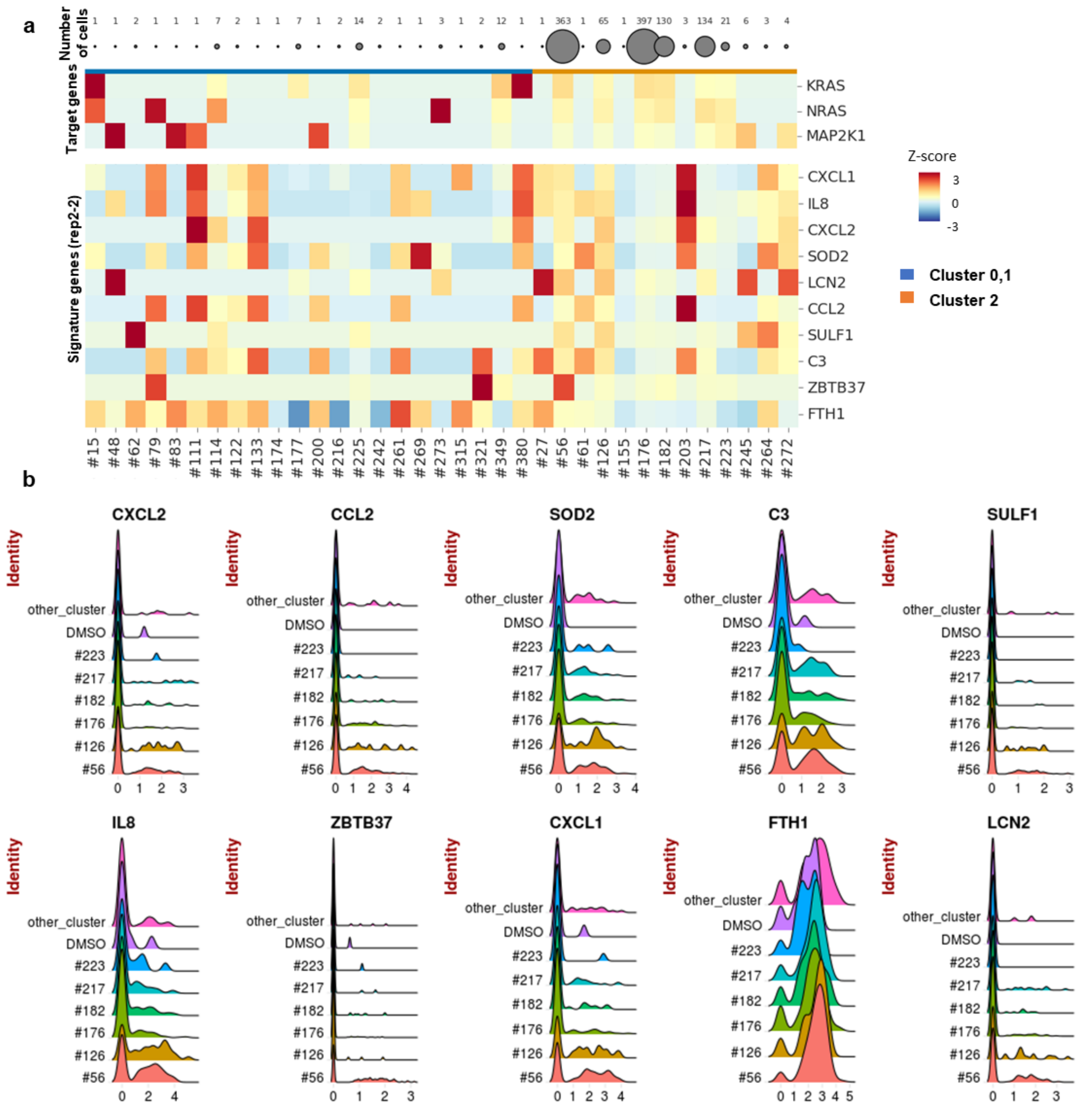


**Supplementary Figure 20 |** Profile of the transcription of signature genes in clusters composed primarily of #56 sgRNA in replicate 2. (**a**) Average expression of signature genes according to individual sgRNA. Each row represents one of the signature genes, and each column represents cells bearing the sgRNA. Cells from Vem(D+28) in other clusters or Vem(D+28) in rep2-2 are arranged from left to right. Color scale indicates the standard deviation of gene expression from the mean expression value, with red indicating high expression and blue indicating low expression. (**b**) Visualization of expression of signature genes in single cells. Vem(D+28) cells in rep2-0 and rep2-1 were grouped into the "other_cluster" category, and DMSO(D+28) cells were grouped into the "DMSO" category. Cells in rep2-2 cluster were individually plotted according to sgRNAs for which the number of cells was >5.

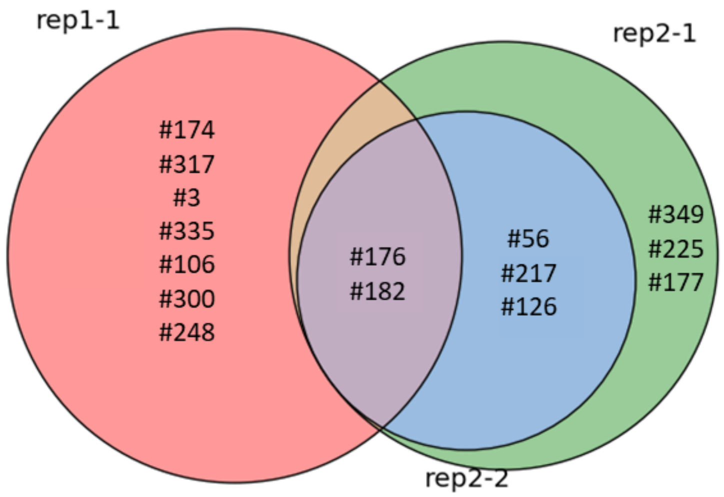


**Supplementary Figure 21 |** Venn diagram of sgRNAs in each cluster that were contained in more than 2 cells.

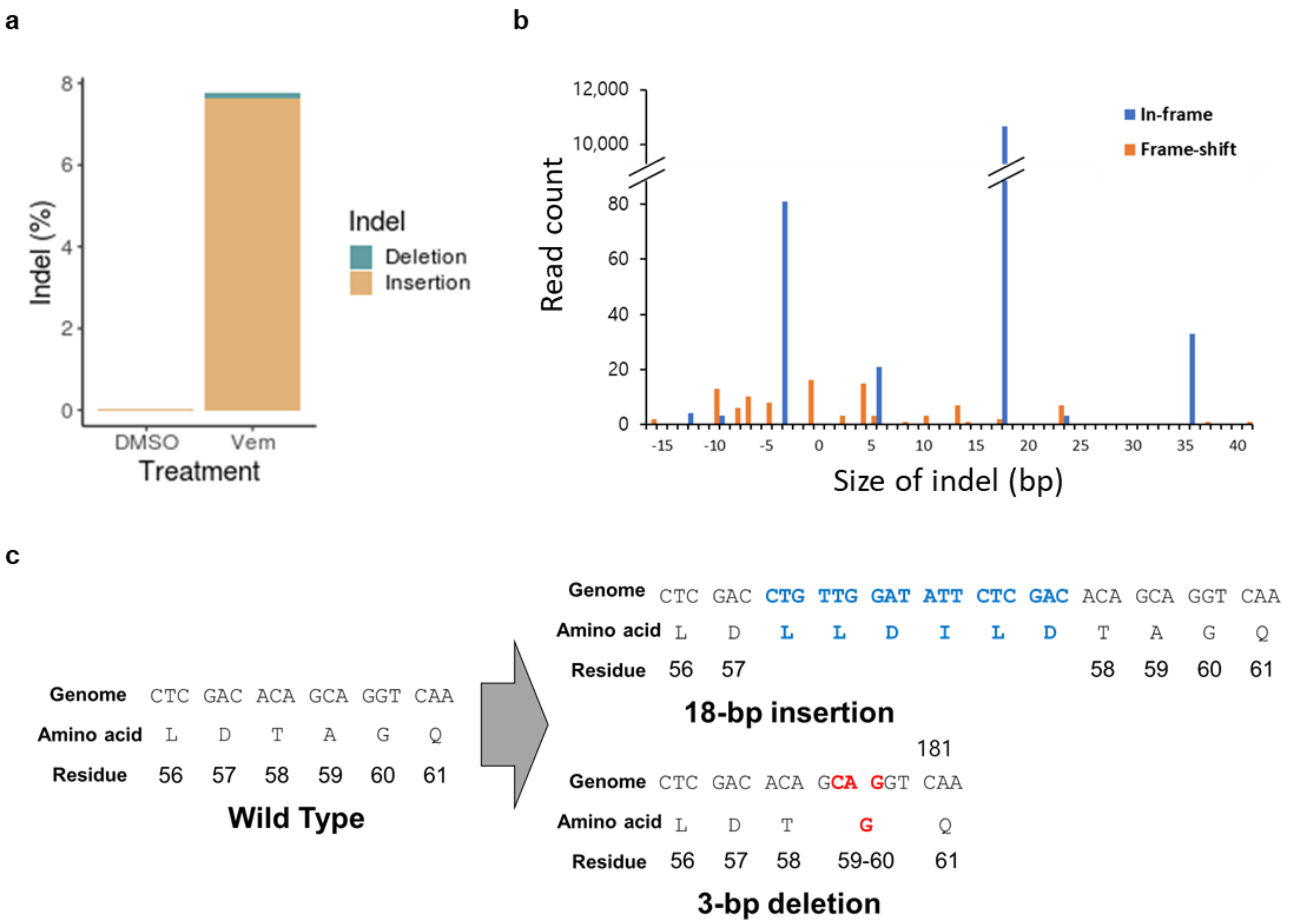


**Supplementary Figure 22 |** Indel mutations found in the target region of the #56 sgRNA in replicate 2. (**a**) Bar graph showing indel frequency in cells treated with DMSO or vemurafenib for 28 days. (**b**) Distribution of read counts of the Indel mutations according to insertion size (positive value) and deletion (negative value). In-frame insertions or deletions are shown in blue, and frame-shift insertions or deletions are shown in orange. (**c**) Two major subtypes of indel mutations. Genome sequence of 18-bp insertion (right upper) is marked in blue, and that of the 3-bp deletion (right lower) is marked in red. Corresponding amino acid and position are shown beneath the genome sequence.

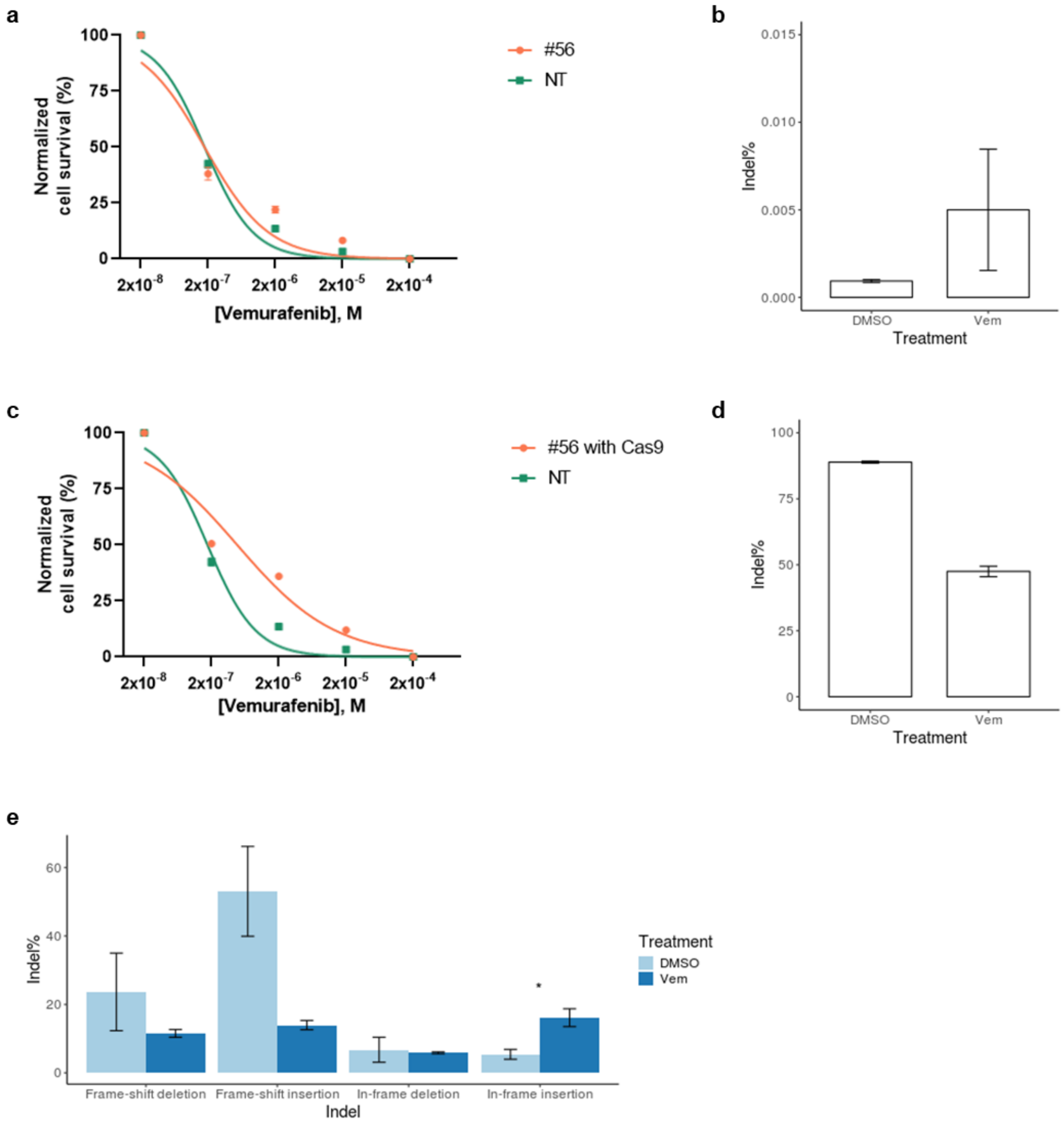


**Supplementary Figure 23 |** Singleplex experiments for #56 sgRNA. (**a**) Vemurafenib dose response curves ($F_{1,26}$ = 0.03237, p = 0.8586, extra sum-of-squares F test between estimated logIC50, n = 3 replicates) and (**b**) bar graph showing indel frequency for cells expressing BE3 and #56 sgRNA. (**c**) Vemurafenib dose response curves ($F_{1,26}$ = 13.94, p = 0.0009, extra sum-of-squares F test between estimated logIC50, n = 3 replicates) and (**d**) bar graph showing indel frequency for cells expressing Cas9 and #56 sgRNA. (**e**) Bar graph showing frame-shift or in-frame indel frequency. p-value for significance of enrichment was determined by one-sided Wilcoxon rank-sum test, *$p < 0.05$. Error bars represent the standard error of mean from three independent experiments.

**a**

| | | | | | | | | | | | | | | | | | | | | PAM | | |
|---|---|---|---|---|---|---|---|---|---|---|---|---|---|---|---|---|---|---|---|---|---|---|
| | G | C | T | G | A | G | A | C | C | C | C | A | C | C | C | A | G | G | C | C | A | A | G |
| **A** | 0.01 | 0.00 | 0.01 | 0.02 | **99.92** | 0.00 | **99.95** | 0.00 | 0.00 | 0.01 | 0.00 | **99.93** | 0.02 | 0.01 | 0.00 | **99.96** | 0.01 | 0.01 | 0.00 | 0.01 | **99.97** | **99.88** | 0.01 |
| **C** | 0.00 | **99.98** | 0.03 | 0.00 | 0.01 | 0.00 | 0.05 | **100.00** | **100.00** | **99.97** | **99.98** | 0.03 | **99.98** | **99.96** | **99.93** | 0.01 | 0.01 | 0.00 | **99.98** | **99.98** | 0.00 | 0.03 | 0.00 |
| **G** | **99.99** | 0.00 | 0.00 | **99.98** | 0.04 | **100.00** | 0.00 | 0.00 | 0.00 | 0.00 | 0.00 | 0.03 | 0.00 | 0.00 | 0.01 | 0.02 | **99.99** | **99.99** | 0.01 | 0.00 | 0.03 | 0.08 | **99.99** |
| **T** | 0.00 | 0.02 | **99.97** | 0.00 | 0.03 | 0.00 | 0.01 | 0.00 | 0.00 | 0.02 | 0.02 | 0.01 | 0.01 | 0.03 | 0.05 | 0.00 | 0.00 | 0.00 | 0.01 | 0.02 | 0.00 | 0.00 | 0.00 |

Vem (D+28)

**b**

| | | | | | | | | | | | | | | | | | | | | PAM | | |
|---|---|---|---|---|---|---|---|---|---|---|---|---|---|---|---|---|---|---|---|---|---|---|
| | T | G | G | T | G | T | T | C | A | A | G | G | T | C | T | C | C | C | A | C | A | A | G |
| **A** | 0.00 | 0.01 | 0.01 | 0.01 | 0.01 | 0.00 | 0.01 | 0.00 | **99.98** | **99.91** | 0.01 | 0.00 | 0.00 | 0.00 | 0.00 | 0.00 | 0.01 | 0.00 | **99.99** | 0.00 | **99.99** | **99.99** | 0.00 |
| **C** | 0.03 | 0.00 | 0.00 | 0.00 | 0.00 | 0.01 | 0.00 | **99.99** | 0.00 | 0.01 | 0.00 | 0.00 | 0.02 | **100.00** | 0.02 | **99.99** | **99.98** | **100.00** | 0.00 | **99.99** | 0.00 | 0.00 | 0.00 |
| **G** | 0.00 | **99.98** | **99.99** | 0.00 | **99.99** | 0.00 | 0.00 | 0.00 | 0.01 | 0.04 | **99.98** | **100.00** | 0.00 | 0.00 | 0.00 | 0.00 | 0.00 | 0.00 | 0.01 | 0.00 | 0.00 | 0.00 | **99.99** |
| **T** | **99.96** | 0.01 | 0.00 | **99.99** | 0.00 | **99.99** | **99.99** | 0.00 | 0.00 | 0.03 | 0.01 | 0.00 | **99.98** | 0.00 | **99.98** | 0.00 | 0.01 | 0.00 | 0.01 | 0.01 | 0.00 | 0.01 | 0.01 |

Vem (D+28)

**Supplementary Figure 24 |** Conversion of C by the #126 and #217 sgRNAs was not observed within the “activity window” in replicate 2. Percentage of sequencing reads with the corresponding base is shown along with the sequence of the protospacer of the #126 (**a**) and #217 (**b**) sgRNAs. Cytosines within the activity window are shown in grey.

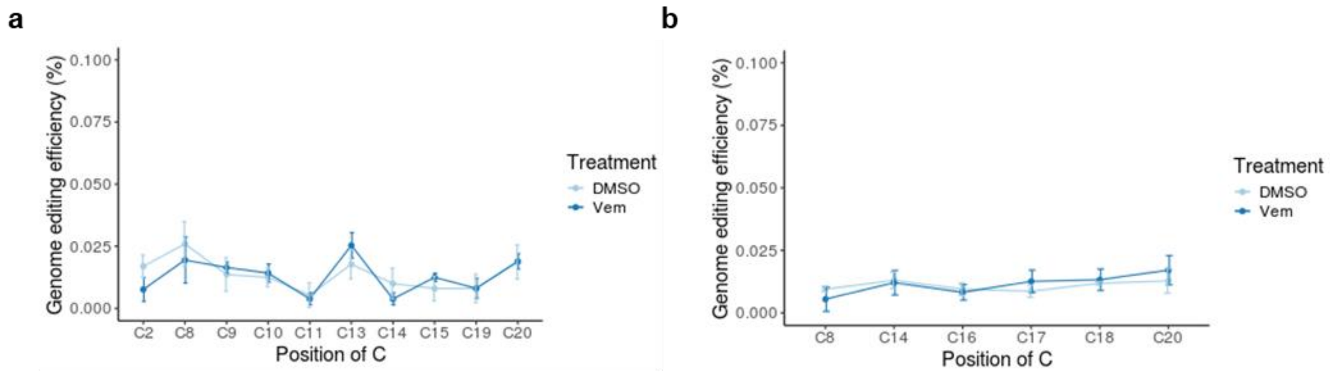


**Supplementary Figure 25 |** Singleplex experiments for #126 and #217 sgRNAs. (**a**) Efficiency of cytosine substitution according to position in the protospacer of the #126 sgRNA. (**b**) Efficiency of cytosine substitution according to position in the protospacer of the #217 sgRNA. All experiments were performed in three independent replicates. Error bars represent the standard error of mean from three independent experiments.

**a**

| rep 1 | | rep 2 | | rep 3 | |
|---|---|---|---|---|---|
| processivity | percent | processivity | percent | processivity | percent |
| CAGGACTTAG | 98.23 | CAGGACTTAG | 98.20 | CAGGACTTAG | 98.22 |
| CAGGATTTAG | 1.24 | CAGGATTTAG | 1.31 | CAGGATTTAG | 1.25 |
| TAGGATTTAG | 0.08 | TAGGATTTAG | 0.08 | TAGGATTTAG | 0.07 |
| TAGGACTTAG | 0.04 | TAGGACTTAG | 0.02 | TAGGACTTAG | 0.03 |

**b**

| rep 1 | | rep 2 | | rep 3 | |
|---|---|---|---|---|---|
| processivity | percent | processivity | percent | processivity | percent |
| CATCCTAGTC | 99.28 | CATCCTAGTC | 99.33 | CATCCTAGTC | 99.22 |
| CATTTTAGTC | 0.19 | CATTTTAGTC | 0.23 | CATTTTAGTC | 0.20 |
| CATTCTAGTC | 0.12 | CATTCTAGTC | 0.07 | CATTCTAGTC | 0.11 |
| CATCTTAGTC | 0.02 | CATCTTAGTC | 0.03 | CATCTTAGTC | 0.02 |
| CATTCTAGTT | 0.00 | CATTCTAGTT | 0.00 | TATCCTAGTC | 0.02 |
| | | | | CATTCTAGTT | 0.00 |

**c**

| rep 1 | | rep 2 | | rep 3 | |
|---|---|---|---|---|---|
| processivity | percent | processivity | percent | processivity | percent |
| CAATACATGA | 95.49 | CAATACATGA | 95.68 | CAATACATGA | 95.72 |
| CAATATATGA | 3.54 | CAATATATGA | 3.54 | CAATATATGA | 3.56 |
| TAATACATGA | 0.03 | TAATACATGA | 0.02 | TAATACATGA | 0.02 |
| | | TAATATATGA | 0.00 | TAATATATGA | 0.01 |

**Supplementary Figure 26 |** Processive deamination was observed in singleplex experiments for (**a**) #25 sgRNA, (**b**) #159 sgRNA, and (**c**) #354 sgRNA. The PAM-distal 10 nt were examined for processivity. Convertible cytidine is underlined, and converted thymine is shown in red font.

## Supplementary Tables

| | D0 | | D+14 | | | | D+28 | | | |
|---|---|---|---|---|---|---|---|---|---|---|
| | initial | | DMSO | | Vemurafenib | | DMSO | | Vemurafenib | |
| | rep1 | rep2 | rep1 | rep2 | rep1 | rep2 | rep1 | rep2 | rep1 | rep2 |
| **Sequenced reads (*10^6)** | 45.01 | 1.47 | 76.29 | 130.59 | 150.65 | 97.54 | 62.37 | 88.50 | 71.47 | 95.67 |
| **Uniquely mapped reads %** | 59.99 | 66.73 | 54.54 | 42.46 | 26.26 | 54.12 | 58.18 | 52.14 | 50.62 | 55.56 |
| **Multiple mapping reads %** | 15.57 | 15.75 | 14.10 | 12.08 | 7.96 | 13.90 | 13.86 | 12.79 | 13.27 | 14.44 |
| **Cells with >500 genes** | 1,606 | 107 | 1,003 | 1,560 | 940 | 2,341 | 627 | 1,543 | 1,531 | 1,960 |
| **Mean unique reads per cell** | 2,260 | 1,614 | 3,534 | 3,811 | 2,289 | 3,114 | 4,959 | 5,125 | 4,032 | 4,048 |
| **Mean genes per cell** | 1,166 | 1,066 | 1,651 | 1,613 | 1,249 | 1,494 | 1,950 | 2,069 | 1,772 | 1,823 |
| **gRNA assigned cells with >500 genes (unique)** | 849 | 83 | 619 | 847 | 516 | 1,252 | 324 | 755 | 971 | 1,212 |
| **Unique gRNA assignment % (cells with >500 genes)** | 52.86 | 77.57 | 61.71 | 54.29 | 54.89 | 53.48 | 51.67 | 48.93 | 63.42 | 61.84 |

**Supplementary Table 1 |** Detailed information regarding the study data. Table shows the characteristics of scRNA-seq data for each step of the study.

| Group | Differentially Expressed Genes | Number of genes overlaped with up-regulated marker genes |
| --- | --- | --- |
| #176 in DMSO | *ABL2, AKT3, AL592183.1, ALKBH3, ASNS, ATF6, ATG12, ATPIF1, ATXN2L, AZI2, BMS1, BPTF, BRCA2, BRD2, C19orf48, C9orf78, CDC16, CEBPB, CELF1, CHD2, CNIH1, COX5B, CTCF, CXCL1, CYB5R2, DDIT4, EFTUD2, EGR1, EHD3, EIF3B, ERC1, ETHE1, FAM105B, FAM204A, FIP1L1, FNBP4, GAR1, GPRC5A, GSS, HCG18, HDAC2, HDAC5, HERC4, HES1, HNRNPU, IL24, IMPA1, INHBA, JUNB, KDM6B, KLF5, LIF, LRAT, MAFG, MAPK1IP1L, MAST4, MED10, METAP2, MPHOSPH10, NAA10, NAA15, NFE2L1, NFKB1, NKTR, NRP2, NUDT1, PABPC1L, PDCL3, PMAIP1, PNPLA6, POLR2J3, PPIH, PSMC5, RAB7A, RBM10, RSF1, RSRC1, RSRC2, RTN4, SEC63, SERBP1, SERTAD4, SETD5, SH2B3, SLC31A1, SMARCA1, SNHG12, SNRPA, SNX10, SRA1, SRRM2, SSR2, STARD4, STRA6, TBCA, TCEA1, TCOF1, THUMPD3, TRAPPC2L, TRIB3, UBTD2, UTP14A, VASP, WDR52, XPA, YARS, ZSWIM6* | *ABL2, C9orf78, HES1, EGR1* |
| #56 in DMSO | *AAMP, ATP5I, ATP5J, C17orf76-AS1, CD44, CKS2, COLGALT1, COX4I1, COX7A2, ERO1L, GABARAPL2, GAS5, GHITM, GTF2F1, GUK1, HSP90B1, HSPA5, LAMC1, MTRNR2L1, NDUFA1, NDUFB10, PAFAH1B2, PAICS, PDCD7, PRPF6, RAB30-AS1, RRBP1, RSF1, SDHB, SLC25A5, SLTM, SNX14, SRSF4, TGFBI, TIMP1, TMEM179B, TPR, TSR1* | |

**Supplementary Table 2 |** Genes that varied under DMSO-treated conditions in cells with #176 and #56 sgRNAs

| Cluster | Gene | p_val | avg_logFC | pct.1 | pct.2 | p_val_adj |
|---|---|---|---|---|---|---|
| **rep1-1** | *CD74* | 2.30E-51 | 1.05203729 | 0.981 | 0.677 | 4.33E-47 |
| | *MALAT1* | 1.43E-49 | 0.63464566 | 1 | 1 | 2.69E-45 |
| | *HLA-DRA* | 1.45E-40 | 1.09659922 | 0.788 | 0.329 | 2.73E-36 |
| | *SLC26A2* | 4.21E-39 | 0.90454379 | 0.947 | 0.717 | 7.94E-35 |
| | *HLA-DRB1* | 1.20E-38 | 0.95840032 | 0.678 | 0.205 | 2.27E-34 |
| | *FOS* | 4.79E-36 | 1.03370216 | 0.774 | 0.314 | 9.03E-32 |
| | *HLA-DPA1* | 1.81E-32 | 0.86955202 | 0.649 | 0.226 | 3.41E-28 |
| | *MAF* | 5.60E-31 | 0.89317038 | 0.529 | 0.139 | 1.06E-26 |
| | *TFAP2B* | 2.10E-29 | 0.98072574 | 0.606 | 0.211 | 3.95E-25 |
| | *PLAT* | 5.35E-29 | 1.13051709 | 0.606 | 0.22 | 1.01E-24 |
| **rep2-1** | *CD74* | 8.87E-54 | 0.99158894 | 0.944 | 0.803 | 1.71E-49 |
| | *HLA-DRA* | 4.62E-53 | 1.186213 | 0.801 | 0.402 | 8.89E-49 |
| | *FOS* | 2.10E-42 | 0.85747235 | 0.846 | 0.484 | 4.05E-38 |
| | *HLA-DRB1* | 9.20E-39 | 0.97022087 | 0.644 | 0.259 | 1.77E-34 |
| | *SPARC* | 3.38E-38 | 0.69862077 | 0.961 | 0.813 | 6.51E-34 |
| | *SLC26A2* | 5.40E-36 | 0.79698574 | 0.896 | 0.726 | 1.04E-31 |
| | *TGFBI* | 1.21E-35 | 0.80869306 | 0.893 | 0.682 | 2.33E-31 |
| | *SEMA3C* | 5.79E-34 | 0.86915942 | 0.769 | 0.461 | 1.11E-29 |
| | *THBS1* | 3.37E-33 | 0.82542133 | 0.733 | 0.375 | 6.50E-29 |
| | *HLA-DPA1* | 5.19E-31 | 0.84924364 | 0.614 | 0.281 | 9.99E-27 |
| **rep1-1** | *CXCL1* | 2.92E-71 | 1.88234591 | 0.784 | 0.177 | 5.63E-67 |
| | *IL8* | 1.16E-66 | 1.96857015 | 0.811 | 0.235 | 2.23E-62 |
| | *CXCL2* | 2.50E-61 | 1.29890299 | 0.584 | 0.077 | 4.82E-57 |
| | *SOD2* | 6.58E-50 | 1.31087496 | 0.668 | 0.177 | 1.27E-45 |
| | *LCN2* | 2.99E-36 | 1.00415193 | 0.547 | 0.135 | 5.75E-32 |
| | *CCL2* | 5.37E-36 | 1.39739539 | 0.521 | 0.121 | 1.03E-31 |
| | *SULF1* | 1.10E-31 | 0.65465475 | 0.363 | 0.057 | 2.11E-27 |
| | *C3* | 5.34E-31 | 0.74342838 | 0.695 | 0.268 | 1.03E-26 |
| | *ZBTB37* | 1.83E-29 | 0.87881432 | 0.489 | 0.135 | 3.52E-25 |
| | *FTH1* | 2.30E-29 | 0.58992827 | 0.984 | 0.861 | 4.43E-25 |

**Supplementary Table 3 |** Signature genes in rep1-1, rep2-1, and rep2-2 obtained from Seurat. Genes are ranked by ascending adjusted p-value.

| Cluster | sgRNA | Count | Cluster | sgRNA | Count | Cluster | sgRNA | Count |
|---|---|---|---|---|---|---|---|---|
| rep1-1 | #176 | 105 | rep2-1 | #176 | 154 | rep2-2 | #56 | 135 |
| | #174 | 29 | | #56 | 48 | | #126 | 19 |
| | #317 | 26 | | #182 | 47 | | #176 | 12 |
| | #3 | 13 | | #217 | 46 | | #182 | 8 |
| | #335 | 8 | | #126 | 11 | | #217 | 4 |
| | #182 | 6 | | #349 | 6 | | #272 | 2 |
| | #106 | 4 | | #225 | 6 | | #61 | 1 |
| | #300 | 3 | | #177 | 3 | | #203 | 1 |
| | #248 | 3 | | #216 | 2 | | #297 | 1 |
| | #169 | 2 | | #272 | 2 | | #223 | 1 |
| | #19 | 2 | | #200 | 1 | | #348 | 1 |
| | #389 | 1 | | #242 | 1 | | #368 | 1 |
| | #410 | 1 | | #155 | 1 | | #155 | 1 |
| | #347 | 1 | | #15 | 1 | | #27 | 1 |
| | #307 | 1 | | #269 | 1 | | #245 | 1 |
| | #56 | 1 | | #380 | 1 | | #264 | 1 |
| | #349 | 1 | | #114 | 1 | | | |
| | #354 | 1 | | #273 | 1 | | | |
| | | | | #48 | 1 | | | |
| | | | | #321 | 1 | | | |
| | | | | #264 | 1 | | | |
| | | | | #122 | 1 | | | |

**Supplementary Table 4 |** sgRNAs in the rep1-1, rep2-1, and rep2-2 clusters. Corresponding counts are shown to the right of each sgRNA.

| sgRNA | Gene | Spacer sequence | Introducible Mutations |
|---|---|---|---|
| #56 | *KRAS* | TCTCGACACAGCAGGTCAAG | L56F, T58I |
| #126 | *MAP2K1* | GCTGAGACCCCACCCAGGCC | A284V, T286I, P287S, P287L, P288L, P288S, P288F, P290L, P290S |
| #217 | *MAP2K1* | TGGTGTTCAAGGTCTCCCAC | S86F, H87Y |

**Supplementary Table 5 |** Cognate mutations of candidate genes.

| Name | Sequence (5' to 3') |
|---|---|
| chip_fwd | GTCCTTCTCAGCCATATAGT |
| chip_rev | GAACACATGTGTCACTCAGT |
| chip_2nd_fwd | TTCTTGGCTTTATATATCTTGTGGAA |
| chip_2nd_rev | GACTAGCCTTATTTTAACTTGCTATTT |
| #176_target_fwd | AAGGGCCTTGGTGTACAGTG |
| #176_target_rev | AGGAGTTGGCCATGGAGTC |
| #126_target_fwd | GAGGTATCCCATCCCTCCTC |
| #126_target_rev | GCATCCAGGGACAGAGAATC |
| #56_target_fwd | CAGGAAGCAAGTAGTAATTGATGG |
| #56_target_rev | ATGGTGAATATCTTCAAATGATTTAGT |
| #217_target_fwd | GGGAGAACTGAAGGATGACG |
| #217_target_rev | TGGTCCCCAGGCTTCTAAGT |
| #25_target_fwd | GAAGAAGGAAGGAAAATTTGGTG |
| #25_target_rev | CCTGTCTTGTCTTTGCTGATG |
| #159_target_fwd (same as #176_target_fwd) | AAGGGCCTTGGTGTACAGTG |
| #159_target_rev (same as #176_target_rev) | AGGAGTTGGCCATGGAGTC |
| #354_target_fwd | TGGTGAAACCTGTTTGTTGG |
| #354_target_rev | TGGTAACCTCATTTCCCCAT |

**Supplementary Table 6 |** Primers used in this study.
