# Supplementary material for: Single-cell analysis of a mutant library generated using CRISPR-guided deaminase in human melanoma cells
Source: Commun Biol. 2020 Apr 2;3:154. doi: 10.1038/s42003-020-0888-2 (PMC7118117; doi:10.1038/s42003-020-0888-2)
Supplement: Supplementary file 2 — Description of Additional Supplementary Files [file 42003_2020_888_MOESM2_ESM.pdf]

Descriptions of additional supplementary files

Supplementary Data 1: Source data

Supplementary Data 2: All possible sgRNAs from all gene isoforms within the human genome.

Supplementary Data 3: 420 sgRNAs designed for all of the exons of *MAP2K1*, *KRAS*, *NRAS*.

Supplementary Data 4: Linear regression results of cells for replicate 1 and 2. Varied genes depend on the condition: treatment, period, and their intersection (treatment:period).

Supplementary Data 5: p-value of ANOVA test according to gene and sgRNA for replicate 1 and 2 result.

Supplementary Data 6: Result of Wilcoxon rank-sum test for DMSO-treated cells.

Supplementary Data 7: Marker genes for the rep1-1, rep2-1 and rep2-2 cluster.

Supplementary Data 8: sgRNAs targeting the same region.
