# Supplementary material for: Single-cell analysis of a mutant library generated using CRISPR-guided deaminase in human melanoma cells
Source: Commun Biol. 2020 Apr 2;3:154. doi: 10.1038/s42003-020-0888-2 (PMC7118117; doi:10.1038/s42003-020-0888-2)
Supplement: Supplementary file 12 — Peer Review File [file 42003_2020_888_MOESM12_ESM.pdf]

Editorial Note: *This manuscript has been previously reviewed at another Nature Research journal. The manuscript was considered suitable for publication without further review at Communications Biology*
